# USP5 Binds and Stabilizes EphA2 to Increase Nasopharyngeal Carcinoma Radioresistance

**DOI:** 10.7150/ijbs.102461

**Published:** 2025-01-06

**Authors:** Jie-Yu Tang, Yun-Xi Peng, Wei Zhu, Jie-Ya Qiu, Wei Huang, Hong Yi, Shan-Shan Lu, Juan Feng, Zheng-Zheng Yu, Di Wu, Qi Wen, Li Yuan, Jinwu Peng, Zhi-Qiang Xiao

**Affiliations:** 1 Department of Pathology, Xiangya Hospital, Central South University, Changsha 410008, China.; 2 Research Center of Carcinogenesis and Targeted Therapy, Xiangya Hospital, Central South University, Changsha 410008, China.; 3 The Higher Educational Key Laboratory for Cancer Proteomics and Translational Medicine of Hunan Province, Xiangya Hospital, Central South University, Changsha, 410008, China.; 4 Department of Nuclear Medicine, The Third Xiangya Hospital, Central South University, Changsha 410013, China.; 5 National Clinical Research Center of Geriatric Disorders, Xiangya Hospital, Central South University, Changsha 410011, China.

**Keywords:** Nasopharyngeal carcinoma, Radioresistance, Mebendazole, USP5, EphA2, Drug repurposing

## Abstract

Radioresistance poses a major challenge in nasopharyngeal carcinoma (NPC) treatment. However, the underlying mechanism of NPC radioresistance remains poorly understood, and the promising radiosensitizer for NPC radiotherapy is also lacked. Overexpression of USP5 and EphA2 has been linked to various cancers, and both the proteins have attracted considerable attention for the development of new anti-cancer drugs. Here, we report that USP5 interacts with EphA2, and increases EphA2 protein stability and expression by ubiquitin proteasome pathway in the NPC cells. Mebendazole (MBZ), a broad-spectrum anthelmintic drug, transcriptionally inhibits USP5 expression, and then promotes EphA2 ubiquitination degradation in the NPC cells. Functionally, USP5 enhances* in vitro* and* in vivo* NPC cell radioresistance via stabilizing EphA2, and MBZ decreases *in vitro* and *in vivo* NPC cell radioresistance via targeting USP5/EphA2 axis. Moreover, the levels of USP5 and EphA2 are significantly higher in the radioresistant NPCs than those in the radiosensitive NPCs, and both proteins for predicting patient prognosis are superior to individual protein. These findings suggest that USP5 binds and stabilizes EphA2 by ubiquitin proteasome pathway to promote NPC radioresistance, and MBZ increases NPC radiosensitivity by targeting USP5/EphA2 axis, and is a potential radiosensitizer in NPC and perhaps in other cancers.

## Introduction

Nasopharyngeal carcinoma (NPC) is a unique subtype of head and neck cancers 1, and is closely associated with the infection of Epstein-Barr virus (EBV) 2. NPC is highly prevalent in southern China and Southeast Asia, which poses one of the most serious public health problems in these areas 1. Radiotherapy is the primary treatment modality for patients with NPC because of its radiosensitive characters. Survival outcomes of patients with NPC improve substantially, mainly benefited from the evolution of radiotherapy techniques and the addition of chemotherapy in patients with loco-regionally advanced disease 3. Nevertheless, radioresistance remains a serious obstacle to successful treatment in many cases. Some of the NPC patients present local recurrences and distant metastases after radiotherapy due to radioresistance and the majority of these patients surrender recurrence and metastasis within 1.5 years after treatment 4, 5. Hence, revealing the molecular mechanism of NPC radioresistance and developing NPC radiosensitization strategy are urgently needed for improving the efficacy and prognosis of NPC patients.

Eph receptors belong to a large family of receptor tyrosine kinases (RTK), and are key regulators of both normal development and disease. Perturbation of Eph receptor and ligand system has been observed in the various human cancers. Particularly, ephrinA2 receptor (EphA2) is the most frequently affected Eph receptor in the human cancers 6. EphA2 is overexpressed in many human cancers, where it promotes tumor growth, metastasis, and stem properties 7-9. As EphA2 is an important oncogenic protein and emerging drug target, approaches for targeting EphA2 downregulation have attracted a considerable interest as anticancer strategies 10. Our previous studies also demonstrated that EphA2 is obviously upregulated in NPC cell lines and tissues, and promotes NPC cell growth, invasion, metastasis and stem properties, and EphA2 expression levels were negatively correlated with NPC patient survival 11-13, suggesting that EphA2 can serve as a therapeutic target in NPC. Recent studies have indicated that EphA2 enhances the radioresistance of lung cancer cells 14, 15, suggesting that EphA2 can serve as a radiosensitization target for tumor radiotherapy.

Ubiquitin proteasome pathway is one of the most common pathways for protein degradation. Following ubiquitin modification, proteins are usually degraded by proteasomes. Correspondingly, there is a deubiquitinating enzyme system that can increase protein stability by removing ubiquitin chains. Accumulated investigations demonstrated that EphA2 can be degraded by ubiquitination proteasome pathway 16, 17. We previously used immunoprecipitation and mass spectrometry analysis (IP-MS) to search proteins interacting with EphA2 in NPC cells and identified deubiquitinating enzyme ubiquitin specific peptidase 5 (USP5) as one of proteins interacting with EphA2 12. However, the function and significance of USP5 and EphA2 interaction are completely unclear.

Drug repurposing, the identification of novel indications for existing and already approved compounds, is becoming attractive as a mean to minimize the chance of failure due to the adverse effects, mitigate the economic load, and accelerate the approval process 18. Mebendazole (MBZ), a broad-spectrum anthelmintic drug, possesses the characteristics of a repurposed drug for treating tumors 19. Numerous investigations suggest that MBZ not only exhibits direct cytotoxic activity for tumor cells 20-24, but also synergizes with ionizing radiation and different chemotherapeutic agents to kill cancer cells 25-27. The recent study indicates that MBZ exhibits antimyeloma activity by transcriptionally inhibiting USP5 expression and then promoting c-Maf protein degradation 28.

In this work, we investigated the effects of USP5 on EphA2 protein stability and NPC cell radiosensitivity, and found that USP5 interacted with EphA2 and decreased its ubiquitination, thereby stabilizing EphA2 in NPC cells, USP5 promoted *in vitro* and* in vivo* NPC cell radioresistance via stabilizing EphA2, and MBZ reduced *in vitro* and* in vivo* NPC cell radioresistance by targeting USP5/EphA2 axis. These findings suggest that USP5/EphA2 axis is a potential novel radiosensitizing target in NPC, and MBZ is a promising radiosensitizer for NPC radiotherapy.

## Materials and Methods

### Patients and tissue specimens

One hundred and nineteen NPC patients without distant metastasis (M0 stage) at the time of diagnosis, who were treated by radical radiotherapy and concurrent chemotherapy according to a uniform guideline in the Xiangya Hospital of Central South University between Jan 2018 and Jan 2020, were recruited in this study. NPC tissue biopsies were obtained from the patients at the time of diagnosis before any therapy, fixed in 4% formalin and embedded in paraffin. We also acquired 24 cases of formalin-fixed and paraffin-embedded normal nasopharyngeal mucosal tissues in the same period, which were used as control. The radiotherapy response was evaluated clinically for primary lesions based on nasopharyngeal fiberscope and magnetic resonance imaging (MRI) one month after the initiation of radiotherapy according to the criteria previously described by us 29, 30. The clinicopathologic features of the patients are presented in Table S1 and S2. For details, see the Supplementary Materials and Methods.

### Animal experiments

Female nude mice (BALB/c nu/nu) that were 4-week old were obtained from the Experimental Animal Center of Central South University and maintained in pathogen-free conditions. The effects of USP5/EphA2 axis and MBZ on NPC cell radiosensitivity were tested in mice. For details, see the Supplementary Materials and Methods.

### Cell lines

Human NPC cell lines HK1 and 5-8F, and HEK293 cell line have been described previously by us 12. Cells were cultured in RPMI-1640 medium supplemented with 10% fetal bovine serum at 37°C in 5% CO_2_. The cell lines were authenticated by short tandem repeat DNA fingerprinting prior to use and were routinely tested negative for mycoplasma contamination using 4,6-diamidino-2-phenylindole staining.

### Establishment of NPC cancer cell lines with expression changes of USP5 or EphA2

Human NPC cell lines were infected with lentiviral vector expressing USP5 shRNA or scramble non-target shRNA (shNC) respectively, and then selected using puromycin for two weeks. Cancer cell lines with the stable knockdown of USP5 and their shNC control cell lines were established. NPC cell lines with the stable knockdown of USP5 were transfected with pcDNA3.1 expressing EphA2 using lipofectamine 2000, and then selected using neomycin for two weeks. Human NPC cell lines with stable USP5 knockdown and EphA2 overexpression were obtained. Human NPC cell lines were transfected with pcDNA3.1 expressing USP5 or EphA2 using lipofectamine 2000, and then selected using neomycin for two weeks. Human NPC cell lines with stable overexpression of exogenous USP5 or EphA2 were obtained. All established NPC cell lines were confirmed by Western blot analysis.

### Immunoprecipitation and immunoblotting

Immunoprecipitation was performed to detect protein-protein interaction and EphA2 ubiquitination. In brief, whole cell lysates were precleared with Protein A/G-Sepharose™ 4B for 2 hours, and then incubated with indicated antibodies or isotype control IgG and Protein A/G-Sepharose™ 4B overnight at 4°C. After 5 times wash with RIPA buffer, beads were boiled in 2×SDS-PAGE loading buffer for 5 minutes to elute protein complexes, followed by SDS-PAGE separation and immunoblotting with specific antibodies.

### GST pull-down assay

The GST pull-down assay was performed to detect USP5 directly interacting with EphA2 as described previously by us 31. For details, see the Supplementary Materials and Methods.

### Western blot

Western blot was performed to detect the expression of proteins in the indicated NPC cells as described previously by us 12, 31. For details, see the Supplementary Materials and Methods.

### Quantitative real-time (qRT)-PCR

QRT-PCR was performed to detect the expression of USP5 and EphA2 in NPC cells with stable knockdown of USP5, NPC cells treated with MBZ and their respective control cells. The primers used are presented in Table S3. For details, see the Supplementary Materials and Methods.

### Dual luciferase reporter assay

A dual luciferase reporter assay was performed to detect USP5 promoter activity in the indicated cells as described previously 31. For details, see the Supplementary Materials and Methods.

### Immunofluorescent staining

Immunofluorescent staining was performed to detect the subcellular location of USP5 and EphA2 and expression of γH2AX in the indicated cells as described previously by us 12, 31. For details, see the Supplementary Materials and Methods.

### Immunohistochemistry and staining evaluation

Immunohistochemistry and staining evaluation of USP5, EphA2, cleaved caspase 3, γH2AX and Ki67 were performed on the formalin-fixed and paraffin-embedded tissue sections as described previously by us 32. For details, see the Supplementary Materials and Methods.

### Molecular docking

The docking model USP5-EphA2 complex was generated using the ClusPro web server for protein-protein docking 33 based on the structures of USP5 (PDB ID: 3IHP) and EphA2 (PDB ID: 1MQB). Structural illustrations were prepared using the PyMOL Molecular Graphic Systems (version 0.99, Schrödinger LLC; http://www.pymol.org/).

### Clonogenic survival assay

A clonogenic survival assay was performed to detect *in vitro* cell sensitivity to ionizing radiation as previously described by us 30. For details, see the Supplementary Materials and Methods.

### Flow cytometric analysis of cell apoptosis

Cell apoptosis was detected using Annexin V*-*APC/7-AAD apoptosis kit as previously described by us 31. For details, see the Supplementary Materials and Methods.

### Statistical analysis

Statistical analysis was performed using IBM SPSS statistical software package 22, and data visualization was generated by GraphPad Prism 8.0. Data are presented as the mean ± standard deviation (SD). For comparisons between two groups, a Student's t-test was used, and for analysis with multiple comparisons, one-way ANOVA, followed by Tukey's post-hoc analysis, was used. Tumor growth curves were assessed by two-way ANOVA with Tukey's post-hoc analysis for multiple comparisons. Correlations were analyzed using the Spearman correlation test. Classification variables were compared by chi-square (χ²) test. Survival curves were obtained by using the Kaplan-Meier method, and comparisons were made by using log-rank test. *P* values <0.05 were considered statistically significant.

## Results

### EphA2 interacts with USP5 in NPC cells

**Figure 1:**
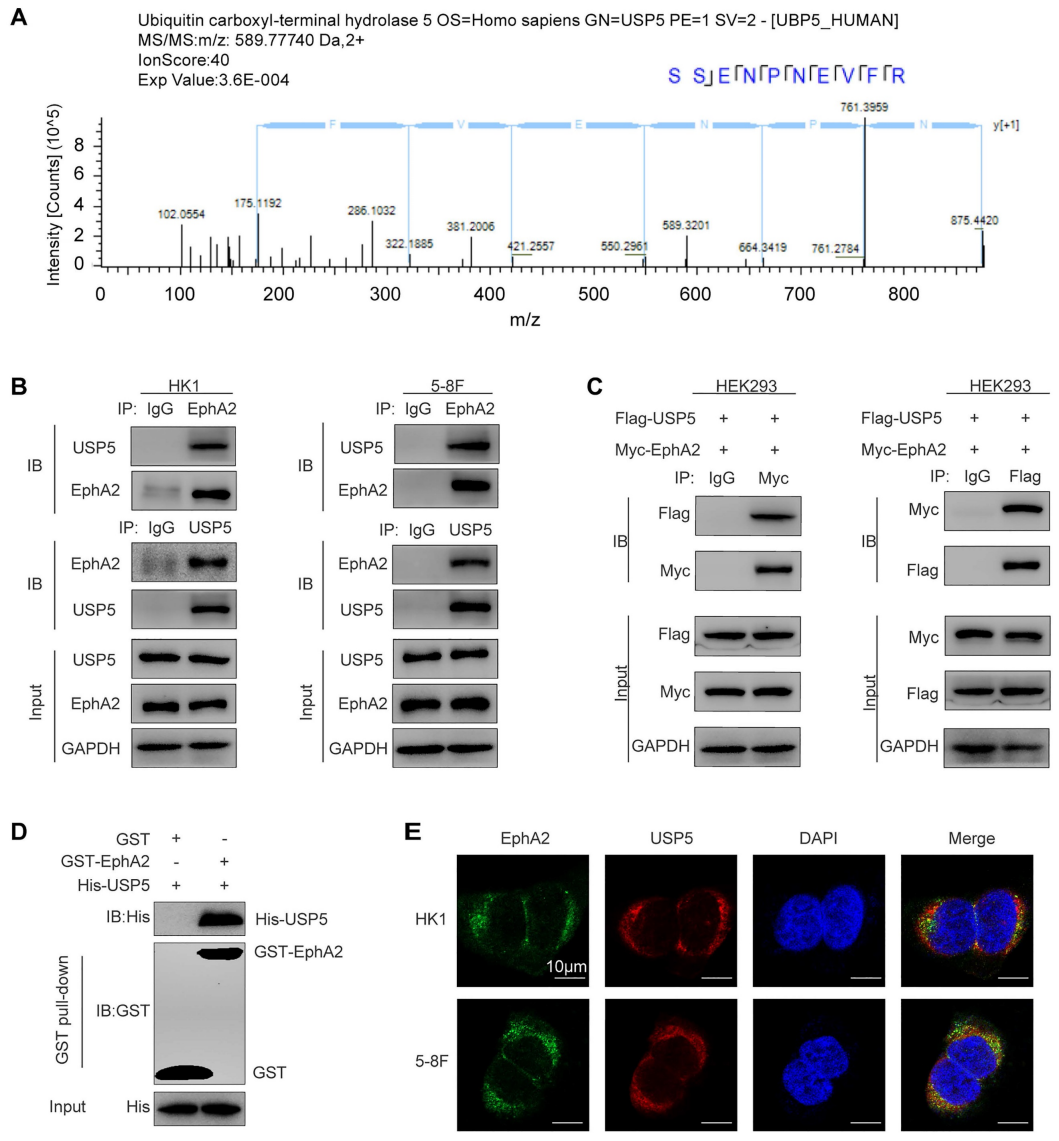
**Identification and validation of USP5 as a protein interacted with EphA2 in NPC cells.** (**A**) Identification of USP5 as a protein interacted with EphA2 by mass spectrometry (MS). The amino acid sequence of a doubly charged peptide with m/z 589.77740 was identified as SSENPNEVFR, and Mascot search showing the peptide matched with USP5. (**B-E**) Validation of USP5 as a protein interacted with EphA2. (**B**) Co-IP showing interaction of endogenous USP5 and EphA2 in the HK1 and 5-8F NPC cells. (**C**) Co-IP showing interaction of exogenous USP5 and EphA2 in the HEK293 cells co-transfected with Flag-USP5 and Myc-EphA2 expression plasmids. (**D**) GST pull-down showing direct interaction of USP5 and EphA2. Purified His tagged USP5 protein was mixed with purified GST or GST tagged EphA2 protein immobilized on glutathione beads. Samples were electrophoresed and immunoblotted with antibodies against GST or Histidine. (**E**) Immunofluorescent staining showing colocalization of EphA2 and USP5 in the HK1 and 5-8F NPC cells. Scale bar, 10 μm. IP, Immunoprecipitation; IB, Immunoblotting.

To comprehensively understand the function of EphA2 in NPC, we previously performed immunoprecipitation and mass spectrometry (IP-MS) to identify the proteins interacting with EphA2 in NPC cells 12. As a result, a total of 1352 proteins including USP5 were identified (Fig. 1A), the proteomic data of which are available via ProteomeXchange with identifier PXD015242. To confirm the interaction of USP5 and EphA2, Co-IP was performed to detect the interaction of USP5 and EphA2, and the results showed USP5 physically interacted with EphA2 in the two NPC cell lines (Fig. 1B), and exogenous USP5 also interacted with exogenous EphA2 in the HEK293 cells co-transfected with USP5 and EphA2 expression plasmids (Fig. 1C). *In vitro* GST pull-down assay using purified proteins showed that USP5 directly interacted with EphA2 (Fig. 1D). Immunofluorescent staining showed that USP5 and EphA2 were colocalized in the NPC cells (Fig. 1E). Collectively, these data provide the evidence for USP5 interacting with EphA2 in NPC cells.

Molecular docking showed that biding of USP5 ZnF-UBP domain (Lys-198 and Asp-203) with EphA2 kinase domain (Asp-841 and Asp-886) (Fig. S1A). To confirm the result of molecular docking, we constructed a series of deletion mutants of USP5 and EphA2 (Fig. S1B), and transfected each of USP5 deletion mutants with wild-type (WT) EphA2 into HEK293 cells following co-IP analysis. The results showed that ZnF-UBP domain deletion (D1-283) USP5 could not bind to EphA2, whereas UBA1 and UBA2 domain deletion (D633-835) USP5 could bind to EphA2 (Fig. S1C). Moreover, USP5 ZnF-UBP domain (1-283) but not USP domain (284-633) and UBA1 and UBA2 domain (634-835) could bind to EphA2 (Fig. S1C), indicating USP5 ZnF-UBP domain (1-283) responsible for binding EphA2. We also transfected each of EphA2 deletion mutants with WT-USP5 into HEK293 cells following co-IP analysis. The results showed that kinase domain deletion (D606-906) EphA2 could not bind to USP5 (Fig. S1D), indicating EphA2 kinase domain (606-906) responsible for binding USP5. These results are in agreement with the results of molecular docking, supporting a model in which USP5 ZnF-UBP domain binds to EphA2 kinase domain.

### USP5 stabilizes EphA2 by ubiquitin proteasome pathway in NPC cells

**Figure 2:**
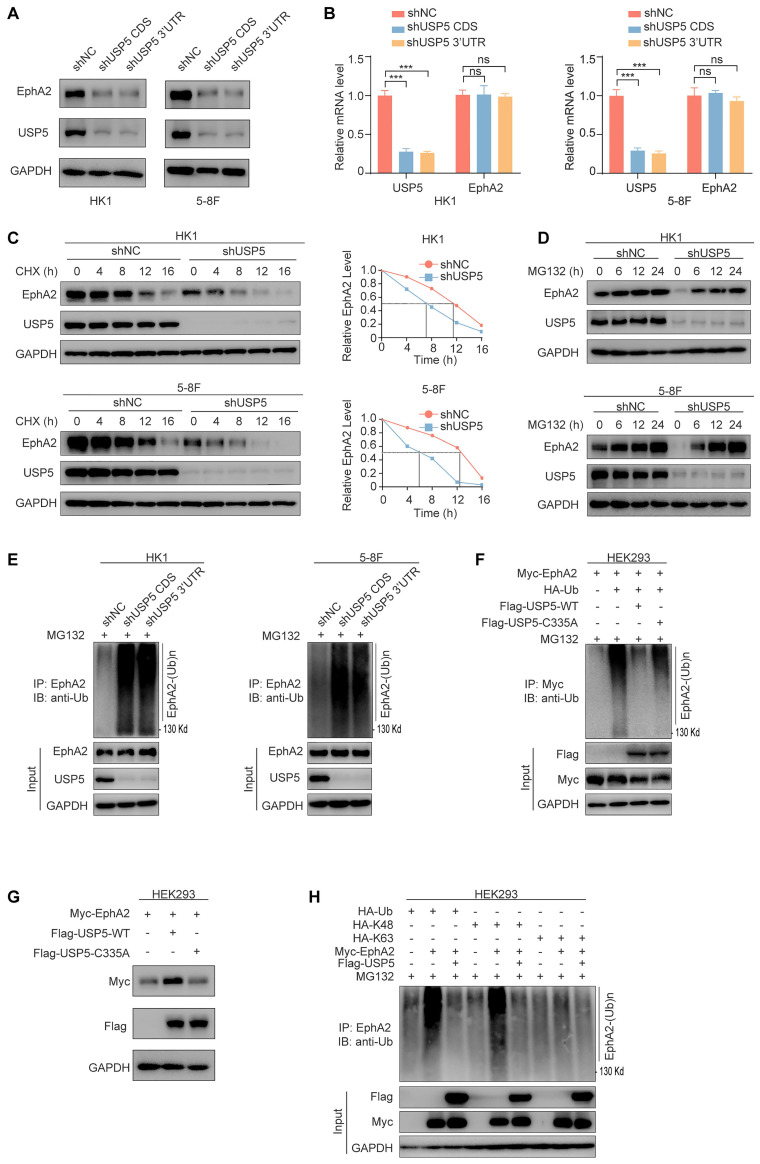
** USP5 stabilizes EphA2 by ubiquitin proteasome pathway in NPC cells.** (**A**) Western blot showing the expression levels of USP5 and EphA2 in the HK1 and 5-8F cells stably transfected with shRNAs against USP5 CDS or 3'UTR, and their shNC control cells. (**B**) QRT-PCR showing the expression levels of USP5 and EphA2 mRNA in the HK1 and 5-8F cells with stable knockdown of USP5. (**C**) Western blot showing the effect of USP5 knockdown on EphA2 protein stability in HK1 and 5-8F NPC cells treated with 20µg/mL cycloheximide (CHX) for indicated times. (**D**) Western blot showing reversion of EphA2 protein levels by proteasome inhibitor MG132 in the HK1 and 5-8F NPC cells with stable knockdown of USP5. Cancer cells were treated with 10 µM MG132 for indicated times. (**E**) USP5 knockdown increases EphA2 polyubiquitination in the HK1 and 5-8F NPC cells. Cancer cells were treated with 10µM MG132 for 12 hours, and subjected to immunoprecipitation analysis with anti-EphA2 antibody followed by immunoblotting with anti-polyubiquitin antibody. (**F**) The effect of wild type USP5 (USP5-WT) and catalytically inactive mutant USP5 (USP5-C335A) on EphA2 polyubiquitination in the HEK293 cells. HEK293 cells were co-transfected with indicated plasmids for 48 hours and treated with 10 µM MG132 for another 12 hours, and subjected to immunoprecipitation analysis with anti-EphA2 antibody followed by immunoblotting with anti-polyubiquitin antibody. (**G**) Western blot showing the effect of USP5-WT and USP5-C335A on EphA2 protein levels in the HEK293 cells co-transfected with indicated plasmids. (**H**) Type of USP5-decreased EphA2 polyubiquitination. HEK293 cells were transfected with indicated plasmids for 48 hours, treated with 10µM MG132 for 12 hours, and subjected to immunoprecipitation analysis with anti-EphA2 antibody followed by immunoblotting with anti-polyubiquitin antibody. Data represent means ± SD. ***, *P* < 0.001; ns, no significance. shUSP5, knockdown of USP5 by shRNA; shNC, scramble shRNA negative control; IP, immunoprecipitation; IB, immunoblotting.

To explore the function of USP5 and EphA2 interaction, we established HK1 and 5-8F NPC cell lines with stable knockdown of USP5 using shRNA and their shNC control cell lines, and observed that knockdown of USP5 significantly downregulated EphA2 protein expression (Fig. 2A), but did not influence EphA2 mRNA levels (Fig. 2B). To confirm USP5-regulated EphA2 expression at the posttranscriptional level, we analyzed the effect of USP5 knockdown on EphA2 protein stability after blocking protein synthesis with cycloheximide (CHX), and observed that EphA2 was rapidly degraded in the USP5 knockdown NPC cells (Fig. 2C), indicating that USP5 tightly controlled EphA2 protein stability. Moreover, the decrease of EphA2 protein in the USP5 knockdown cells was specifically reversed by treatment with the proteasome inhibitor MG132 (Fig. 2D), but not reversed by treatment with the lysosome inhibitor chloroquine (CQ) (Fig. S2A), indicating a proteasome-dependent mechanism in EphA2 destabilization by USP5 knockdown.

Considering USP5 as a DUB, we analyzed whether USP5 affects the stability of EphA2 by deubiquitinating EphA2. Our findings revealed that the reduction of USP5 levels in NPC cancer cells led to an elevation in the ubiquitination of EphA2 (Fig. 2E), and wild-type USP5 (USP5-WT) also decreased exogenous EphA2 ubiquitination levels, and upregulated its protein levels in the HEK293 cells cotransfected with USP5 and EphA2 expression plasmids (Fig. 2F and G), but catalytically inactive mutant USP5-C335A 34 almost did not influence the ubiquitination and protein levels of exogenous EphA2 in the HEK293 cells (Fig. 2F and G). We also observed that USP5-WT but not USP5-C335A reduced the ubiquitination levels of endogenous EphA2 in the USP5 knockdown HK1 and 5-8F NPC cells (Fig. S2B). Moreover, we examined the composition of the USP5-inhibited EphA2 polyubiquitin chains. As shown in Fig. 2H, the total and K48-linked ubiquitination but not the K63-linked ubiquitination of EphA2 was obviously downregulated by USP5, suggesting that USP5 inhibits the K48-linked polyubiquitination of EphA2. Together, these results indicate that USP5 increases EphA2 protein stability by ubiquitin proteasome pathway in the NPC cells.

### Mebendazole degrades EphA2 via inhibiting USP5-mediated EphA2 deubiquitination in NPC cells

**Figure 3:**
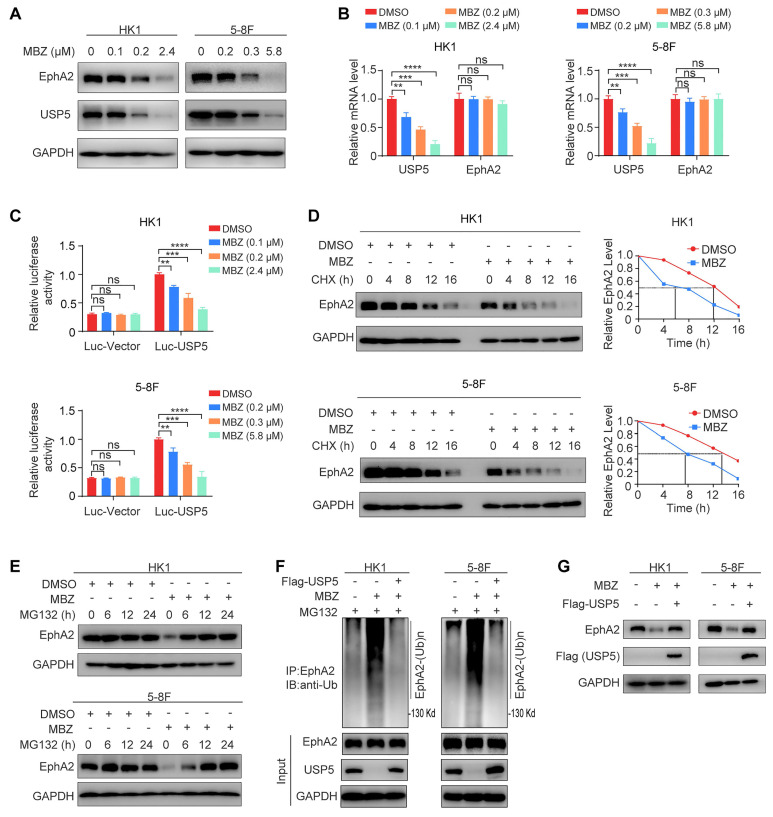
** Mebendazole degrades EphA2 via inhibiting USP5-mediated EphA2 deubiquitination in NPC cells.** Western blot (**A**) and qRT-PCR (**B**) showing the protein and mRNA levels of USP5 and EphA2 in the HK1 and 5-8F NPC cells treated with indicated concentrations of MBZ for 48 hours. (**C**) Dual-luciferase reporter gene assay showing the effect of MBZ on USP5 promoter activity in the HK1 and 5-8F NPC cells. The luciferase reporter plasmid driven by USP5 promoter or control plasmid and pRL-TK plasmid were co-transfected into cancer cells for 12 hours. 36 hours after treated with indicated concentrations of MBZ, USP5 reporter activity was assessed by using the Dual-luciferase reporter assay system. (**D**) MBZ decreases EphA2 protein stability in the HK1 and 5-8F NPC cells. Cancer cells were treated with 2.4 µM (HK1 cells) or 5.8 µM (5-8F) for 36 hours, followed by treatment with 20µg/mL CHX for indicated times, and subjected to western blot analysis with anti-EphA2 antibody. (**E**) Western blot showing reversion of EphA2 protein levels by proteasome inhibitor MG132 in the HK1 and 5-8F NPC cells with MBZ. Cancer cells were treated with 2.4 µM (HK1 cells) or 5.8 µM (5-8F) for 36 hours, followed by treatment with 10 µM MG132 for indicated times. (**F**) USP5 overexpression reverses EphA2 polyubiquitination levels in the NPC cells treated with MBZ. HK1, 5-8F NPC cells and their respective USP5 overexpression cells were treated with IC50 of MBZ, and subjected to immunoprecipitation analysis with anti-EphA2 antibody followed by immunoblotting with anti-polyubiquitin antibody. (**G**) USP5 overexpression reverses EphA2 protein levels in the NPC cells treated with MBZ. HK1, 5-8F NPC cells and their respective USP5 overexpression cells were treated with IC50 of MBZ, and subjected to western blot analysis with anti-EphA2 antibody. Data represent means ± SD. **, *P* < 0.01; ***, *P* < 0.001; ****, *P* < 0.0001; ns, no significance.

Our results have demonstrated that USP5 stabilizes EphA2 by binding and removing its ubiquitination (Fig. 2). Previous study has indicated that mebendazole (MBZ) downregulates USP5 expression by suppressing USP5 transcription in myeloma cells 28. Thus, we sought to determine the effect of MBZ on USP5/EphA2 axis in NPC cells. For this purpose, HK1 and 5-8F NPC cell lines were treated with MBZ for 24 hours at increasing concentrations, and cell viability was detected using the MTT assay and half maximal inhibitory concentrations (IC50) were calculated. The results showed that the IC50 of HK1 and 5-8F cells treated with MBZ was 2.4 μM and 5.8 μM respectively (Fig. S3A), and Western blot also showed that MBZ decreased EphA2 expression in a time-dependent manner (Fig. S3B). Next, we detected the USP5 and EphA2 levels in the HK1 and 5-8F NPC cell lines treated with MBZ for 48 hours. Western blot analysis showed that MBZ downregulated the protein expression of both USP5 and EphA2 in the two NPC cell lines in a dose-dependent manner (Fig. 3A). QRT-PCR showed that MBZ downregulated USP5 mRNA but not EphA2 mRNA expression in the two NPC cell lines (Fig. 3B), suggesting that MBZ downregulates USP5 expression by suppressing USP5 transcription in NPC cells. We also performed a dual luciferase reporter assay to determine the effect of MBZ on USP5 promoter activity. The results showed that MBZ significantly decreased the USP5 promoter activity of the two NPC cell lines in a dose-dependent manner (Fig. 3C), supporting that MBZ transcriptionally suppresses USP5 expression in the NPC cells. To confirm MBZ-downregulated EphA2 expression at the posttranscriptional level, we analyzed the effect of MBZ on EphA2 protein stability after blocking protein synthesis with CHX, and observed that EphA2 was rapidly degraded in the MBZ-treated NPC cells (Fig. 3D), indicating that MBZ decreased EphA2 protein stability. Moreover, the decrease of EphA2 protein in the MBZ-treated NPC cells was specifically reversed by treatment with MG132 (Fig. 3E), suggesting that MBZ induces EphA2 degradation via a ubiquitin proteasome pathway. Next, we evaluated the effect of MBZ on the ubiquitination and expression levels of EphA2, and observed that MBZ significantly increased EphA2 ubiquitination levels, and decreased EphA2 expression levels in the NPC cancer cells (Fig. 3F and G). Moreover, overexpression of exogenous USP5 could antagonize the effect of MBZ on the ubiquitination and expression levels of EphA2 (Fig. 3F and G). These results suggest that MBZ degrades EphA2 via inhibiting USP5-mediated EphA2 deubiquitination, and USP5/EphA2 axis is a potential target of MBZ in the NPC cells.

### USP5 increases *in vitro* and* in vivo* NPC cell radioresistance via stabilizing EphA2

**Figure 4:**
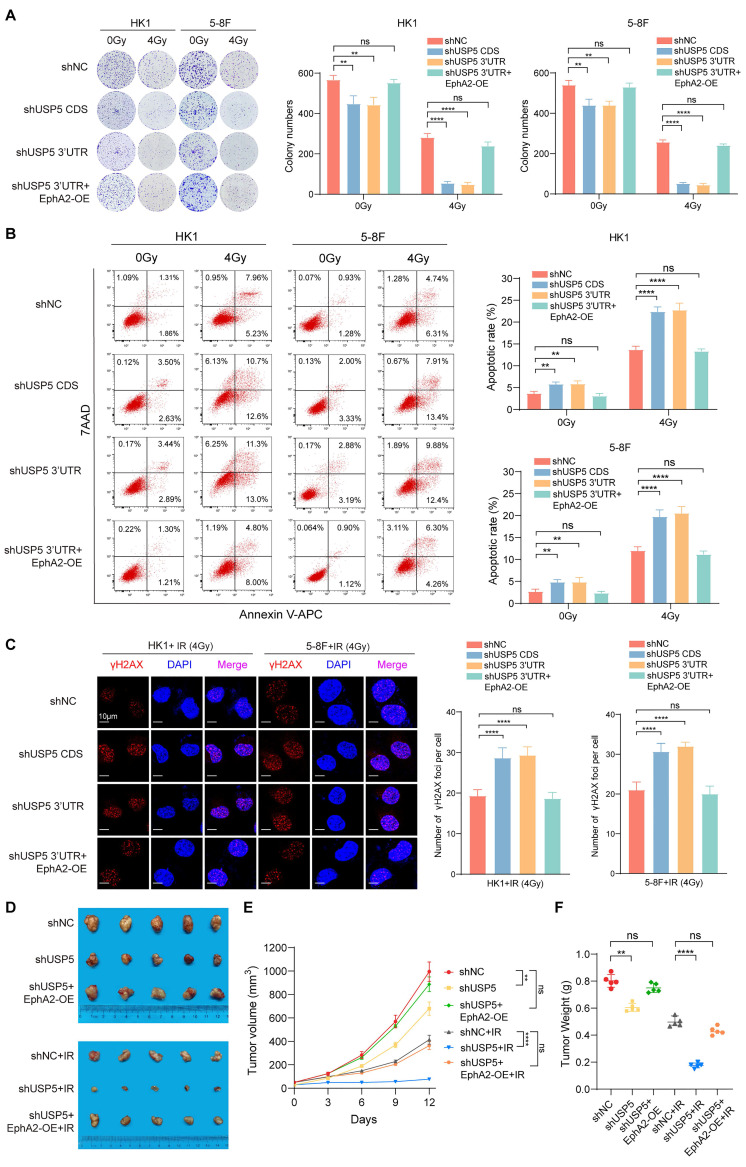
** USP5 increases *in vitro* and *in vivo* NPC cell radioresistance through EphA2 mediation.** (**A**) Clonogenic survival assay showing the radiosensitivity of HK1 and 5-8F NPC cells with USP5 knockdown or USP5 knockdown and EphA2 overexpression (OE). Cancer cells were exposed to 4 Gy ionizing radiation (IR), and colonies that formed after incubation of 12 days were stained with crystal violet. Representative images are shown on the left, and quantitative data are presented on the right. Cells without irradiation served as control. (**B**) Flow cytometric analysis showing the irradiation-induced apoptotic rate of HK1 and 5-8F NPC cells with USP5 knockdown or USP5 knockdown and EphA2 OE. 48 hours after 4Gy IR, cell apoptosis was detected. Representative results are shown on the left, and quantitative data are presented on the right. Cells without irradiation served as control. (**C**) Immunofluorescent staining showing the irradiation-induced γH2AX focus number of HK1 and 5-8F NPC cells with USP5 knockdown or USP5 knockdown and EphA2 OE. 4 hours after 4Gy IR, γH2AX foci were detected. Representative images are shown on the left, and quantitative data are presented on the right. (**D**) Xenografts showing the *in vivo* radiosensitivity of 5-8F NPC cells with USP5 knockdown, 5-8F NPC cells with USP5 knockdown and EphA2 overexpression and shNC control cells. The photographs (*left*), growth curves (*middle*) and weight (*right*) of xenograft tumors 12 days after initial radiotherapy. 7 days after the inoculation, tumor-bearing mice received a total of 12Gy IR, and radioresponse was estimated after initial treatment. Xenografts without irradiation served as control. shUSP5, knockdown of USP5 by shRNA; EphA2-OE, EphA2 overexpression. **, *P* < 0.01; ****, *P* < 0.0001; ns, no significance.

USP5 plays a crucial role in the tumor development and progression, and is a potential target for cancer therapy 28, 35-37. However, whether USP5 regulates tumor radiosensitivity is unclear. Therefore, we evaluated the effects of USP5 on NPC cell radiosensitivity. We established NPC cell lines with USP5 knockdown, NPC cell lines with USP5 knockdown and EphA2 overexpression, and their shNC control cell lines (Fig. S4A). A clonogenic survival assay showed that USP5 knockdown significantly decreased the colony formation ability of NPC cells in both 4Gy ionizing radiation (IR) and no irradiation groups, but the inhibitory effect of USP5 knockdown on colony formation ability was stronger in 4Gy IR group than in no irradiation group, and EphA2 overexpression recovered the colony formation ability of NPC cells with USP5 knockdown in both groups (Fig. 4A). Irradiation primarily leads to double-strand DNA breaks (DSBs), and unrepaired or misrepaired DSBs in the DNA lead to cell apoptosis. The apoptosis resulting from irradiation is, to a considerable degree, understood as radiosensitivity 38. Therefore, we analyzed the effect of USP5 knockdown on the irradiation-induced apoptosis of NPC cells. Flow cytometric analysis showed that USP5 knockdown significantly increased apoptosis of NPC cells in both 4Gy IR and no irradiation groups, but the promotion effect of USP5 knockdown on cell apoptosis was stronger in 4Gy IR group than in no irradiation group, and EphA2 overexpression recovered the apoptosis of NPC cells with USP5 knockdown in both groups (Fig. 4B). Moreover, immunofluorescent staining of γH2AX, a marker of DSBs, showed that USP5 knockdown alone did not result in γH2AX focus formation in the NPC cells (data not shown), whereas USP5 knockdown significantly increased irradiation-induced γH2AX focus number in the NPC cells, and EphA2 overexpression recovered irradiation-induced γH2AX focus number in the NPC cells with USP5 knockdown (Fig. 4C). Taken together, these results demonstrate that USP5 increases *in vitro* NPC cell radioresistance via EphA2.

To determine the effect of USP5 on *in vivo* NPC cell radiosensitivity, we generated subcutaneous tumors in nude mice using 5-8F NPC cells with USP5 knockdown, 5-8F NPC cells with USP5 knockdown and EphA2 overexpression, and shNC control 5-8F cells respectively (Fig. S4B). Seven days after the inoculation, a total of 12 Gy IR was delivered to the tumor, tumor radioresponse in mice was assessed. The results showed that USP5 knockdown inhibited growth of xenografted tumors in both IR and no irradiation mice, but the inhibitory effect of USP5 knockdown on xenografted tumors was stronger in IR mice than in no irradiation mice, and EphA2 overexpression could antagonize the inhibitory effect of USP5 knockdown on xenografted tumors in both IR and no irradiation mice (Fig. 4D).

Moreover, we detected the expression of cleaved caspase 3 (indicator of cell apoptosis), γH2AX (a marker of DSBs), and Ki67 (index of cell proliferation) in the xenografted tumors by immunohistochemistry (IHC), and observed that USP5 knockdown increased the expression of cleaved caspase 3, and decreased Ki67 expression in the xenografted tumors of both IR and no irradiation mice, but the effect of USP5 knockdown on the two protein expression was stronger in the irradiated tumors than in no irradiation tumors, and EphA2 overexpression could antagonize the effect of USP5 knockdown on the two proteins expression in both irradiated and no irradiation tumors (Fig. S5). We also observed that USP5 knockdown alone did not affect γH2AX expression in the xenografted tumors, whereas USP5 knockdown increased irradiation-induced γH2AX expression in the xenografted tumors, and EphA2 overexpression recovered irradiation-induced γH2AX expression in the xenografted tumors with USP5 knockdown (Fig. S5). Taken together, our results demonstrate that USP5 increases *in vivo* NPC cell radioresistance via EphA2. Our data also suggest that targeting USP5/EphA2 axis has a potential for NPC radiosensitization.

### Mebendazole decreases *in vitro* and *in vivo* NPC cell radioresistance via targeting USP5/EphA2 axis

**Figure 5:**
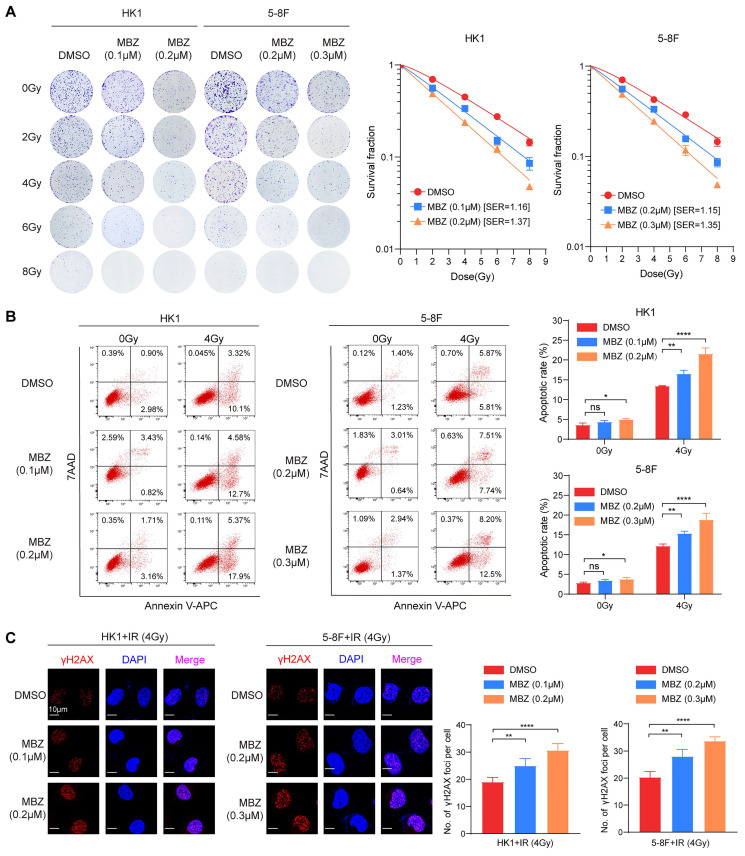
** MBZ increases *in vitro* NPC cell radiosensitivity.** (**A**) Clonogenic survival assay showing the effects of MBZ on the radiosensitivity of HK1 and 5-8F NPC cells. Cells were treated with indicated concentration of MBZ for 48 hours, followed by IR (0-8Gy). Colonies that formed after incubation of 12 days were stained with crystal violet and photographed (*left*), and sensitive enhancement ratio (SER) of MBZ was calculated (*right*). (**B**) Flow cytometric analysis showing the effects of MBZ on irradiation-induced apoptosis of HK1 and 5-8F NPC cells. Cancer cells were treated with indicated concentration of MBZ for 48 hours, followed by 4Gy IR. 48 hours after IR, cell apoptosis was detected. Representative results are shown on the left, and quantitative data are presented on the right. Cells without irradiation served as control. (**C**) Immunofluorescent staining showing the effects of MBZ on irradiation-induced γH2AX focus number of HK1 and 5-8F NPC cells. Cancer cells were treated with indicated concentration of MBZ for 48 hours, followed by 4Gy IR. 4 hours after IR, γH2AX foci were detected. Representative images are shown on the left, and quantitative data are presented on the right. *, *P* < 0.05; **, *P* < 0.01; ****, *P* < 0.0001; ns, no significance.

USP5/EphA2 axis plays a critical role in promoting NPC cell radioresistance (Fig. 4), and MBZ inhibits USP5/EphA2 axis in NPC cells (Fig. 3). Therefore, we sought to determine the effects of MBZ on NPC cell radiosensitivity. For this purpose, HK1 and 5-8F NPC cells were treated with low concentrations (IC10 and IC20) of MBZ for 48 hours, and then were exposed to gradually increasing doses of IR (1-8Gy). A clonogenic survival assay was performed to test whether MBZ has a radiosensitizing effect on NPC cells. As shown in Fig. 5A, MBZ significantly radiosensitized NPC cells in a dose-dependent manner, as demonstrated by a sensitizer enhancement ratio (SER). Moreover, HK1 and 5-8F NPC cells were treated with low concentration MBZ (IC10 and IC20) of MBZ for 48 hours, and followed by exposure to 4Gy IR. Flow cytometric analysis showed that low concentrations of MBZ have a light effect on NPC apoptosis, whereas cell apoptotic rate was significantly higher in the cells treated with a combination of MBZ and IR than in the cells treated with IR alone (Fig. 5B). Immunofluorescent staining of γH2AX foci showed that low concentrations (IC10 and IC20) of MBZ did not result in DSBs (data not shown), whereas γH2AX focus number was significantly higher in the NPC cells treated with a combination of MBZ and IR than in the NPC cells treated with IR alone (Fig. 5C). These results indicate that MBZ increases *in vitro* NPC cell radiosensitivity.

**Figure 6:**
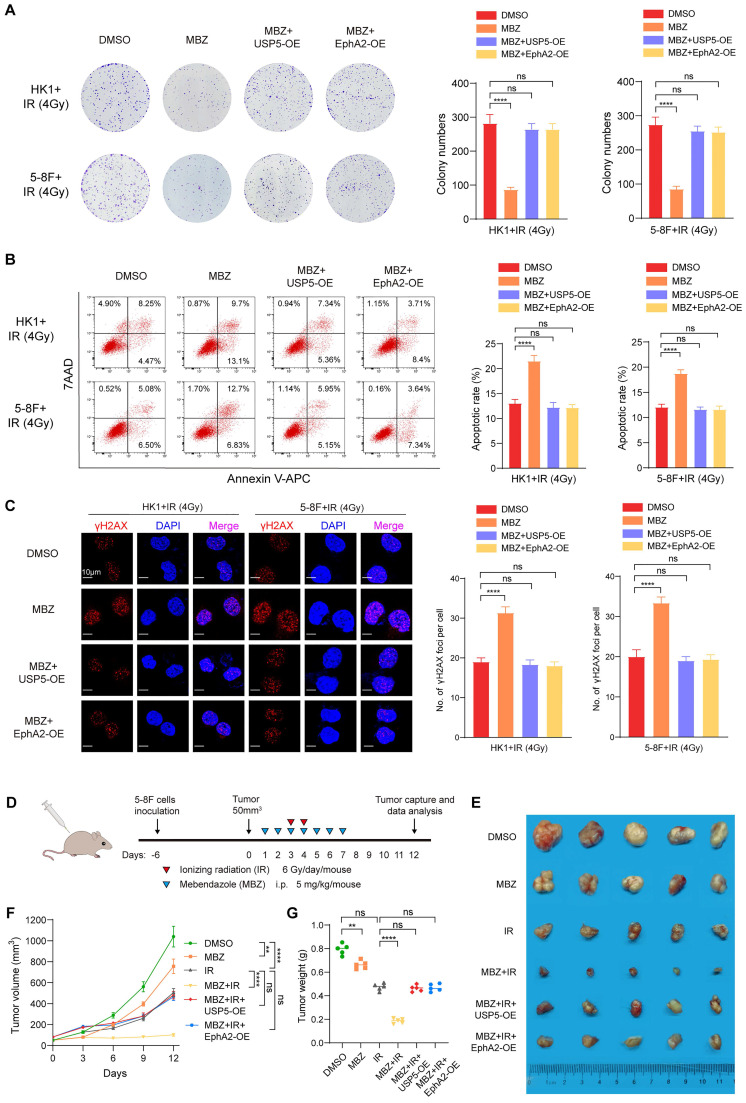
** Mebendazole increases NPC cell radiosensitivity via targeting USP5/EphA2 axis.** (**A**) USP5 or EphA2 overexpression (OE) reverses the effect of MBZ on colonic formation ability in HK1 and 5-8F NPC cells treated with IR. Cancer cells were treated with IC50 of MBZ for 48 hours, followed by 4Gy IR, and subjected to clonogenic survival assay. Colonies that formed after incubation of 12 days were stained with crystal violet. Representative images are shown on the left, and quantitative data are presented on the right. (**B**) USP5 or EphA2 OE reverses the effect of MBZ on irradiation-induced apoptosis of HK1 and 5-8F NPC cells treated with IR. Cancer cells were treated with IC50 of MBZ for 48 hours, followed by 4Gy IR. 48 hours after IR, cell apoptosis was detected by flow cytometry. (**C**) USP5 or EphA2 OE reverses the effect of MBZ on γH2AX focus number of HK1 and 5-8F NPC cells treated with IR. Cancer cells were treated with MBZ for 48 hours, followed by 4Gy IR. 4 hours after IR, γH2AX foci were detected by immunofluorescent staining. (**D-E**) MBZ enhances *in vivo* NPC cell radiosensitivity via inhibiting USP5/EphA2 axis. A schematic diagram illustrating the treatment plan of MBZ or/and IR in the nude mice with xenografts generated from 5-8F NPC cells (**D**). The photographs of xenograft tumors 12 days after initial treatment (**E**). The growth curves of xenograft tumors after initial treatment (**F**). Summary of weight data of xenograft tumors 12 days after initial treatment (**G**). **, *P* < 0.01; ****, *P* < 0.0001; ns, no significance.

To explore whether MBZ radiosensitizes NPC cells via targeting USP5/EphA2 axis, we established HK1 and 5-8F NPC cell lines with stable overexpression of USP5 or EphA2 and their vector control cell line. These established cell lines were treated with MBZ and 4Gy IR, and subjected to radiosensitive analyses. A clonogenic survival assay showed that either EphA2 or USP5 overexpression recovered radioresistance of NPC cells treated with MBZ (Fig. 6A). Both flow cytometric analysis of cell apoptosis and immunofluorescent staining of γH2AX foci showed that either EphA2 or USP5 overexpression antagonized irradiation-induced cell apoptosis and γH2AX focus formation of NPC cells treated with MBZ (Fig. 6B and C). The results demonstrate that MBZ increases *in vitro* NPC cell radiosensitivity via targeting USP5/EphA2 axis.

To test the effect of MBZ on *in vivo* NPC cell radiosensitivity, 5-8F cells with USP5 or EphA2 overexpression and vector control 5-8F cells were subcutaneously inoculated into nude mice respectively. Seven days after inoculation, the tumor-bearing mice received treatment of MBZ (5 mg/kg once daily for 7 days) via peritoneal injection and/or a total of 12 Gy IR as indicated in Fig. 6D. Twelve days after initial treatment, we assessed tumor suppression function of MBZ and/or IR. As shown in Figure 6E-G, treatment of the tumor-bearing mice with MBZ or IR significantly inhibited the growth of tumors generated from vector control 5-8F NPC cells, the combination of MBZ with IR treatment had stronger tumor suppression compared with IR alone, and either EphA2 or USP5 overexpression antagonized radiosensitizing effect of MBZ on the xenografted tumors. Moreover, we detected the expression of cleaved caspase 3, γH2AX, and Ki67 in the xenografted tumors treated with MBZ or/and IR by IHC. The results showed that MBZ or IR increased cleaved caspase 3 expression, and decreased Ki67 expression, and the combination of MBZ with IR treatment had stronger effect on the two protein expression, and either EphA2 or USP5 overexpression antagonized the effect of MBZ on the two protein expression (Fig. S6). Moreover, MBZ alone did not affect γH2AX expression in the xenografted tumors, whereas IR increased γH2AX expression, and the combination of MBZ with IR treatment had stronger effect on γH2AX expression, and either EphA2 or USP5 overexpression antagonized the effect of MBZ on γH2AX expression (Fig. S6). Taken together, the results suggest that MBZ enhances *in vivo* NPC cell radiosensitivity via inhibiting USP5/EphA2 axis.

### USP5 and EphA2 expression levels are correlated with NPC radiosensitivity and patient prognosis

**Figure 7:**
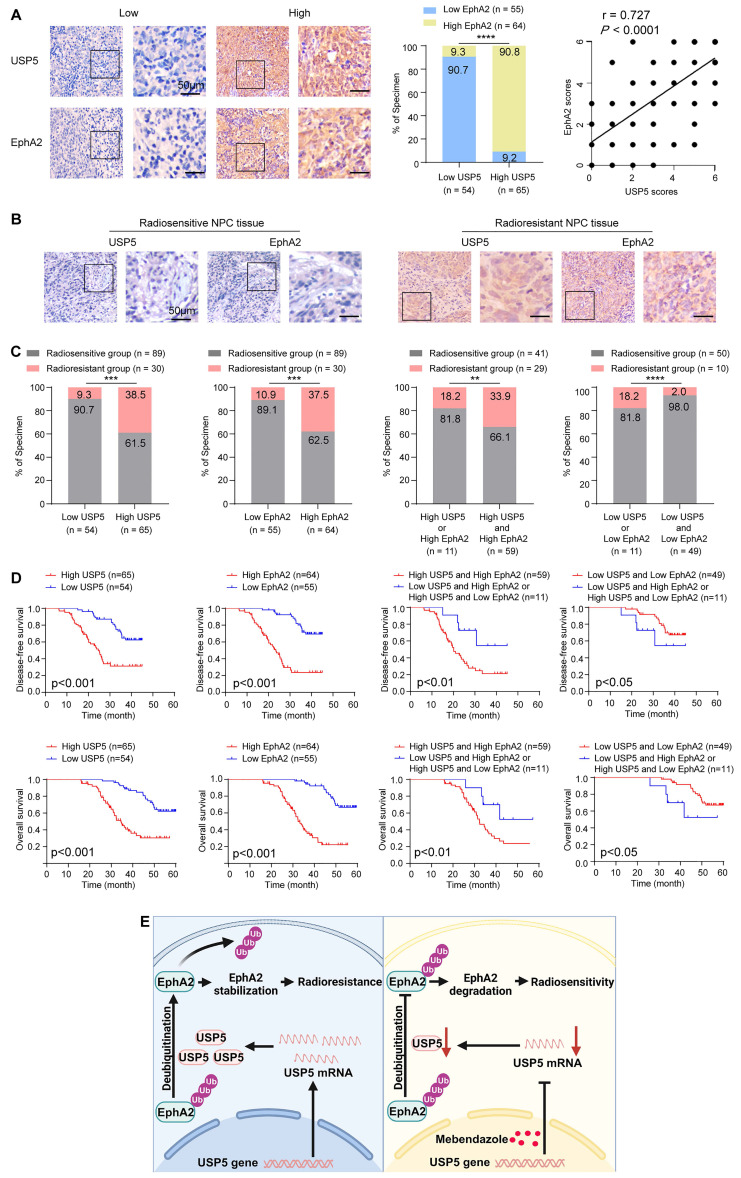
** Expression levels of USP5 and EphA2 correlate with NPC radiosensitivity and patient prognosis.** (**A**) Positive correlation between alterations for USP5 and EphA2 expressions in NPC tissues. Representative IHC images of low and high expression of USP5 and EphA2 in NPC tissues are shown on the left, and statistical analysis of the correlation between USP5 and EphA2 expressions is presented on the right (*P*<0.001, Spearman correlation test). (**B**) Representative IHC images of USP5 and EphA2 expression in the radiosensitive and radioresistant NPC tissues. (**C**) Histograms showing the proportion of radiosensitive and radioresistant NPCs based on the expression levels of USP5, EphA2, or both proteins. Statistical differences were determined by χ2 test. (**D**) Survival analysis of the NPC patients. Kaplan-Meier survival analysis of disease-free survival (*top*) and overall survival (*bottom*) for 119 NPC patients based on the expression levels of USP5, EphA2 or both proteins. Log-rank test was used to calculate P value. (**E**) A model for radiosensitization of Mebendazole by targeting USP5/EphA2 axis in NPC. In NPC cells, USP5 binds and stabilize EphA2 by inhibiting its ubiquitinaion degradation, promoting NPC cell radioresistance (*left*). Mebendazole increases NPC cell radiosensitivity by transcriptionally inhibiting USP5 expression and then targeting EphA2 degradation (*right*). Scale bars = 50 μm. **, *P* < 0.01; ***, *P* < 0.001; ****, *P* < 0.0001.

To investigate the clinical significance of USP5/EphA2 axis, we used IHC to detect expression levels of USP5 and EphA2 in 119 tumor tissues from patients with NPC, who received radical radiotherapy and concurrent chemotherapy according to a uniform guideline. 24 normal nasopharyngeal mucosal tissues were used as control. The clinicopathological characteristics of the patients are shown in Table S1. IHC showed that expression levels of both USP5 and EphA2 in the cancer tissues were significantly higher than those in the normal tissues (Fig. S7), and USP5 levels were positively correlated with EphA2 levels in cancer tissues (Fig. 7A), supporting USP5 as the deubiquitinase of EphA2 in NPC.

We further assessed correlation of USP5 and EphA2 expression levels with NPC radiosensitivity in these patients. In the 119 patients, 30 and 89 patients were classified as radioresistant and radiosensitive patients. The expression levels of USP5/EphA2 were significantly higher in the radioresistant NPCs than those in the radiosensitive NPCs (Fig. 7B and C, and Table S2), and were correlated with lymphnode metastasis and TNM stage (Table S2). Moreover, the ability of USP5 and EphA2 in distinguishing radiosensitive and radioresistant NPCs was analyzed by determining the ROC curves of the two proteins individually and as a panel. The area under curve values of the two proteins are listed in Table S4 together with their individual and collective values of merit. Sensitivity and specificity of USP5 and EphA2 combination (a panel) in discriminating radiosensitive from radioresistant NPCs are significantly higher than those of individual protein (Fig. S8, and Table S4), indicating that USP5 and EphA2 might serve as a biomarker for predicting NPC radiosensitivity.

Since radioresistance is a major cause that leads to the poor outcomes of NPC patients, we analyzed the ability of USP5 and EphA2 to predict disease free survival (DFS) and overall survival (OS) of the patients. Survival analysis showed that patients with high levels of USP5/EphA2 had a shorter DFS and OS relative to patients with low levels of USP5/EphA2 (Fig. 7D). Moreover, patients with high levels of both USP5 and EphA2 had a shorter DFS and OS relative to patients with high level of one protein alone (Fig. 7D). A univariate and multivariate Cox regression analysis showed that a combination of USP5 and EphA2 was an independent predictor for both DFS and OS (Table S5). Together, these data indicate that a combination of USP5 and EphA2 could be considered as a marker for predicting prognosis in patients with NPC.

## Discussion

In this study, we found that USP5 binds and stabilizes EphA2 by removing its polyubiquitination in NPC cells. USP5 plays an oncogenic role in various human cancers 28, 35-37. Our previous studies have revealed that EphA2 is a potential therapeutic target for NPC 11-13, and recent studies have indicated that EphA2 increases radioresistance of lung cancer cells 14, 15. Therefore, we explored whether USP5 stabilizes EphA2 to promote NPC radioresistance, and found that USP5 increases *in vitro* and *in vivo* NPC cell radioresistance via stabilizing EphA2, indicating that USP5/EphA2 axis promotes NPC cell radioresistance. To our knowledge, it is for the first time reported that USP5 promotes tumor radioresistance.

Stabilization of EphA2 by USP5 is important for increasing NPC cell radioresistance *in vitro* and in mice. Consistent with this observation, our IHC showed that the expression levels of USP5/EphA2 were significantly higher in the radioresistant NPCs than those in the radiosensitive NPCs, indicating that USP5-stabilizing EphA2 might increase clinical NPC radioresistance. Survival analysis showed that patients with high levels of USP5/EphA2 had a shorter DFS and OS relative to patients with low levels of USP5/EphA2. Notably, NPC patients with the high level of both EphA2 and USP5 proteins had higher radioresistance, and poorer DFS and OS relative to the patients with the high level of one protein alone, indicating that a combination of USP5 and EphA2 could serve as an important marker for predicting NPC patient prognosis, and guiding personalized NPC radiotherapy.

USP5 stabilizes EphA2 to increase NPC cell radioresistance, indicating that inhibition of USP5/EphA2 axis may become a promising strategy for NPC radiosensitization. MBZ, a broad-spectrum anthelmintic drug, not only transcriptionally inhibits USP5 expression in myeloma cells 28, but also has efficacy against different types of solid tumors 20-24, which is a repurposed drug for treating tumors.

Therefore, we investigated whether MBZ sensitizes NPC cells to ionizing radiation (IR) by targeting USP5/EphA2 axis. Firstly, we analyzed the effect of MBZ on USP5/EphA2 axis in NPC cells, and observed that MBZ transcriptionally inhibits USP5 expression and then degrades EphA2 protein via increasing its ubiquitination. Next, we assessed *in vitro* and *in vivo* radiosensitizing effects of MBZ on NPC cells, and found that MBZ obviously increases radiosensitivity in NPC cell lines and their xenografted tumors, indicating that MBZ can serve as a radiosensitizer in NPC radiotherapy. This is the first evidence that MBZ sensitizes NPC cells to ionizing radiation* in vitro* and *in vivo*. We also observed that USP5 or EphA2 overexpression recovers radioresistance of NPC cells and their xenografted tumors treated with MBZ, indicating that MBZ sensitizes NPC cells to ionizing radiation by targeting USP5/EphA2 axis.

One of underlying reasons for radioresistance is attributable to the presence of cancer stem cells (CSCs) inside tumors, which are responsible for metastases, relapses, radiotherapy failure, and a poor prognosis in cancer patients 39. Zhang *et al.* found that MBZ radiosensitizes triple-negative breast cancer (TNBC) cells by preventing radiation-induced de-differentiation of TNBC cells into CSCs 40. MBZ dose-dependently decreases the fraction of ALDH1 positive cancer cells, resulting in a significant depletion of CSCs capable of self-renewal 25. Accumulating studies have revealed that both USP5 and EphA2 promote cancer stemness 11, 40, 41. These studies suggest that radiosensitizing effect of MBZ might be related to decreases of CSCs inside NPC by targeting USP5/EphA2 axis. In addition, anti-tumor activity of MBZ is involves in targeting various pathways and molecules, which depends on the cancer type and context. For an example, MBZ can increase tumor radiosensitivity by inhibiting the accumulation of DNA damage response proteins, NBS1 and CHK2, in the cell nucleus independently of the induction of mitotic arrest 42, indicating that MBZ sensitizes cancer cells to IR by inhibiting DNA damage repair. Whether MBZ NPC radiosensitization is involved in DNA damage repair needs further investigation.

Our results demonstrate that MBZ increases NPC cell radiosensitivity. MBZ has a very favorable toxicity profile in humans even at high doses administered over lengthy periods of time 22. Moreover, MBZ was also well-tolerated in a phase II clinical trial of recurrent glioblastoma 43. Considering the low toxicity in humans and NPC radiosensitizing effect of MBZ in mice, it is worth further proceeding MBZ to clinical trial for NPC radiosensitization. Moreover, the results presented in this study also add to the existing body of literature supporting the repurposing of MBZ for treatment of cancers.

In summary (Fig. 7E), our present study identifies USP5 as a novel interactor of EphA2, and USP5 binds and stabilizes EphA2 via ubiquitin proteasome pathway in NPC cells, and demonstrates that USP5/EphA2 axis promotes NPC radioresistance, and MBZ has an obvious radiosensitizing effect on NPC by targeting USP5/EphA2 axis.

These data suggest that MBZ is a potential radiosensitizer in NPC and perhaps in other cancers.

## Supplementary Material

Supplementary materials and methods, figures and tables.

## References

[1] Chen YP, Chan ATC, Le QT, Blanchard P, Sun Y, Ma J (2019). Nasopharyngeal carcinoma. Lancet.

[2] Young LS, Yap LF, Murray PG (2016). Epstein-Barr virus: more than 50 years old and still providing surprises. Nat Rev Cancer.

[3] Sun Y, Li WF, Chen NY, Zhang N, Hu GQ, Xie FY (2016). Induction chemotherapy plus concurrent chemoradiotherapy versus concurrent chemoradiotherapy alone in locoregionally advanced nasopharyngeal carcinoma: a phase 3, multicentre, randomised controlled trial. Lancet Oncol.

[4] Lee AW, Poon YF, Foo W, Law SC, Cheung FK, Chan DK (1992). Retrospective analysis of 5037 patients with nasopharyngeal carcinoma treated during 1976-1985: overall survival and patterns of failure. Int J Radiat Oncol Biol Phys.

[5] Leung SF, Teo PM, Shiu WW, Tsao SY, Leung TW (1991). Clinical features and management of distant metastases of nasopharyngeal carcinoma. J Otolaryngol.

[6] Pasquale EB (2010). Eph receptors and ephrins in cancer: bidirectional signalling and beyond. Nat Rev Cancer.

[7] Markosyan N, Li J, Sun YH, Richman LP, Lin JH, Yan F (2019). Tumor cell intrinsic EPHA2 suppresses anti-tumor immunity by regulating PTGS2 (COX-2). J Clin Invest.

[8] Zhou Y, Yamada N, Tanaka T, Hori T, Yokoyama S, Hayakawa Y (2015). Crucial roles of RSK in cell motility by catalysing serine phosphorylation of EphA2. Nat Commun.

[9] Miao H, Li DQ, Mukherjee A, Guo H, Petty A, Cutter J (2009). EphA2 mediates ligand-dependent inhibition and ligand-independent promotion of cell migration and invasion via a reciprocal regulatory loop with Akt. Cancer cell.

[10] Xiao T, Xiao Y, Wang W, Tang YY, Xiao Z, Su M (2020). Targeting EphA2 in cancer. J Hematol Oncol.

[11] Li JY, Xiao T, Yi HM, Yi H, Feng J, Zhu JF (2019). S897 phosphorylation of EphA2 is indispensable for EphA2-dependent nasopharyngeal carcinoma cell invasion, metastasis and stem properties. Cancer lett.

[12] Feng J, Lu SS, Xiao T, Huang W, Yi H, Zhu W (2020). ANXA1 binds and stabilizes EphA2 to promote nasopharyngeal carcinoma growth and metastasis. Cancer Res.

[13] Xiang YP, Xiao T, Li QG, Lu SS, Zhu W, Liu YY (2020). Y772 phosphorylation of EphA2 is responsible for EphA2-dependent NPC nasopharyngeal carcinoma growth by Shp2/Erk-1/2 signaling pathway. Cell Death Dis.

[14] Graves PR, Din SU, Ashamalla M, Ashamalla H, Gilbert TSK, Graves LM (2017). Ionizing radiation induces EphA2 S897 phosphorylation in a MEK/ERK/RSK-dependent manner. Int J of Radiat Biol.

[15] Gong S, Li Y, Lv L, Men W (2022). Restored microRNA-519a enhances the radiosensitivity of non-small cell lung cancer via suppressing EphA2. Gene Ther.

[16] Walker-Daniels J, Riese DJ 2nd, Kinch MS (2002). c-Cbl-dependent EphA2 protein degradation is induced by ligand binding. Mol Cancer Res.

[17] Naudin C, Sirvent A, Leroy C, Larive R, Simon V, Pannequin J (2014). SLAP displays tumour suppressor functions in colorectal cancer via destabilization of the SRC substrate EPHA2. Nat Commun.

[18] Cha Y, Erez T, Reynolds IJ, Kumar D, Ross J, Koytiger G (2018). Drug repurposing from the perspective of pharmaceutical companies. Br J Pharmacol.

[19] Guerini AE, Triggiani L, Maddalo M, Bonù ML, Frassine F, Baiguini A (2019). Mebendazole as a candidate for drug repurposing in oncology: an extensive review of current literature. Cancers (Basel).

[20] Bai RY, Staedtke V, Aprhys CM, Gallia GL, Riggins GJ (2011). Antiparasitic mebendazole shows survival benefit in 2 preclinical models of glioblastoma multiforme. Neuro Oncol.

[21] Bai RY, Staedtke V, Rudin CM, Bunz F, Riggins GJ (2015). Effective treatment of diverse medulloblastoma models with mebendazole and its impact on tumor angiogenesis. Neuro Oncol.

[22] Larsen AR, Bai RY, Chung JH, Borodovsky A, Rudin CM, Riggins GJ (2015). Repurposing the antihelmintic mebendazole as a hedgehog inhibitor. Mol Cancer Ther.

[23] Doudican N, Rodriguez A, Osman I, Orlow SJ (2008). Mebendazole induces apoptosis via Bcl-2 inactivation in chemoresistant melanoma cells. Mol Cancer Res.

[24] Nygren P, Fryknäs M, Agerup B, Larsson R (2013). Repositioning of the anthelmintic drug mebendazole for the treatment for colon cancer. J Cancer Res Clin Oncol.

[25] Zhang L, Bochkur Dratver M, Yazal T, Dong K, Nguyen A (2019). Mebendazole potentiates radiation therapy in triple-negative breast cancer. Int J Radiat Oncol Biol Phys.

[26] Skibinski CG, Williamson T, Riggins GJ (2018). Mebendazole and radiation in combination increase survival through anticancer mechanisms in an intracranial rodent model of malignant meningioma. J Neurooncol.

[27] Kipper FC, Silva AO, Marc AL, Confortin G, Junqueira AV, Neto EP (2018). Vinblastine and antihelmintic mebendazole potentiate temozolomide in resistant gliomas. Invest New Drugs.

[28] Chen XH, Xu YJ, Wang XG, Lin P, Cao BY, Zeng YY (2019). Mebendazole elicits potent antimyeloma activity by inhibiting the USP5/c-Maf axis. Acta Pharmacol Sin.

[29] Feng XP, Yi H, Li MY, Li XH, Yi B, Zhang PF (2010). Identification of biomarkers for predicting nasopharyngeal carcinoma response to radiotherapy by proteomics. Cancer Res.

[30] Qu JQ, Yi HM, Ye X, Zhu JF, Yi H, Li LN (2015). MiRNA-203 reduces nasopharyngeal carcinoma radioresistance by targeting IL8/AKT signaling. Mol Cancer Ther.

[31] Xiao D, Zeng T, Zhu W, Yu ZZ, Huang W, Yi H (2023). ANXA1 promotes tumor immune evasion by binding PARP1 and upregulating Stat3-induced expression of PD-L1 in multiple cancers. Cancer Immunol Res.

[32] Yi HM, Yi H, Zhu JF, Xiao T, Lu SS, Guan YJ (2016). A five-variable signature predicts radioresistance and prognosis in nasopharyngeal carcinoma patients receiving radical radiotherapy. Tumour Biol.

[33] Kozakov D, Hall DR, Xia B, Porter KA, Padhorny D, Yueh C (2017). The ClusPro web server for protein-protein docking. Nat Protoc.

[34] Cai B, Zhao J, Zhang Y, Liu Y, Ma C, Yi F (2022). USP5 attenuates NLRP3 inflammasome activation by promoting autophagic degradation of NLRP3. Autophagy.

[35] Xu X, Huang A, Cui X, Han K, Hou X, Wang Q (2019). Ubiquitin specific peptidase 5 regulates colorectal cancer cell growth by stabilizing Tu translation elongation factor. Theranostics.

[36] Ning F, Xin H, Liu J, Lv C, Xu X, Wang M (2020). Structure and function of USP5: Insight into physiological and pathophysiological roles. Pharmacol Res.

[37] Meng J, Ai X, Lei Y, Zhong W, Qian B, Qiao K (2019). USP5 promotes epithelial- mesenchymal transition by stabilizing SLUG in hepatocellular carcinoma. Theranostics.

[38] Gudkov AV, Komarova EA (2003). The role of p53 in determining sensitivity to radiotherapy. Nat Rev Cancer.

[39] Rycaj K, Tang DG (2014). Cancer stem cells and radioresistance. Int J Radiat Biol.

[40] Tung CH, Wu JE, Huang MF, Wang WL, Wu YY, Tsai YT (2023). Ubiquitin-specific peptidase 5 facilitates cancer stem cell-like properties in lung cancer by deubiquitinating β-catenin. Cancer Cell Int.

[41] Binda E, Visioli A, Giani F, Lamorte G, Copetti M, Pitter KL (2012). The EphA2 receptor drives self-renewal and tumorigenicity in stem-like tumor-propagating cells from human glioblastomas. Cancer Cell.

[42] Markowitz D, Ha G, Ruggieri R, Symons M (2017). Microtubule-targeting agents can sensitize cancer cells to ionizing radiation by an interphase-based mechanism. Onco Targets Ther.

[43] Patil VM, Menon N, Chatterjee A, Tonse R, Choudhari A, Mahajan A (2022). Mebendazole plus lomustine or temozolomide in patients with recurrent glioblastoma: A randomised open-label phase II trial. EClinicalMedicine.

