# Supplementary material for: USP5 Binds and Stabilizes EphA2 to Increase Nasopharyngeal Carcinoma Radioresistance
Source: Int J Biol Sci. 2025 Jan 6;21(3):893–909. doi: 10.7150/ijbs.102461 (PMC11781186; doi:10.7150/ijbs.102461)
Supplement: Supplementary file 1 — Supplementary materials and methods, figures and tables. [file ijbsv21p0893s1.pdf]

Supplementary Material

Supplementary Materials and Methods

Supplementary Table S1-5

Supplementary Figure S1-8

Supplementary Materials and Methods

Patients and tissue specimens

One hundred and nineteen NPC patients without distant metastasis (M0 stage) at the time of diagnosis, who were treated by radical radiotherapy and concurrent chemotherapy according to a uniform guideline in the Xiangya Hospital of Central South University between Jan 2018 and Jan 2020, were recruited in this study. NPC tissue biopsies were obtained from these patients at the time of diagnosis before any therapy, fixed in 4% formalin and embedded in paraffin. We also acquired 24 cases of formalin-fixed and paraffin-embedded normal nasopharyngeal mucosal tissues in the same period, which were used as control. On the basis of the 2017 WHO classification, all tumors were histopathologically diagnosed as undifferentiated non-keratinizing squamous cell carcinomas. The TNM stage of the patients was classified and reclassified according to the 2017 AJCC staging system.

The radiotherapy response was evaluated clinically for primary lesions based on nasopharyngeal fiberscope and MRI one month after the initiation of radiotherapy according to the following criteria as described previously by us [1, 2]. Radioresistant NPC patients were defined as ones with persistent disease (incomplete regression of primary tumor and/or neck lymphonodes) at >3 months or with local recurrent disease at the nasopharynx and/or neck lymphonodes at ≤12 months after completion of radiotherapy. Radiosensitive NPC patients were defined as ones without the local residual lesions (complete regression) at >3 months and without local recurrent disease at >12 months after completion of radiotherapy. Distant metastasis was excluded by skeletal, thoracic, and upper abdominal imaging before radiotherapy. On the basis of the above criteria, 119 NPC patients comprised 89 radiosensitive and 30 radioresistant ones.

The patients were followed up, and the follow-up period at the time of analysis was 6 to 100 months (average 40.70±13.34). Overall survival (OS) was defined as the time from the initiation of treatment to the date of cancer-related death or when censored at the latest date if patients were still alive. Disease-free survival was calculated as the time from the completion of treatment to the date of pathological diagnosis or clinical evidence of local failure and/or distant metastasis. Detailed clinicopathologic features of the patients are presented in Tables S1 and S2.

Antibodies and reagents

Antibodies used in this study included USP5 (sc-390943, Santa Cruz), USP5 (A4202, Abclonal), EphA2 (sc-398832, Santa Cruz), GAPDH (AC002, Abclonal), Myc-tag (AE010, Abclonal), Flag-Tag (AE092, Abclonal), HA-Tag (66006-2-Ig, Proteintech), His-Tag (AE003, Abclonal), GST-Tag (SAB4301139, Sigma-Aldrich), Ubiquitin (91112, CST), γH2AX (2577, CST), cleaved caspase 3 (9661, CST), Ki67 (sc-23900, Santa Cruz), HRP anti-rabbit IgG (7074, CST), HRP anti-mouse IgG (7076, CST), Rabbit control IgG (3900, CST), Mouse control IgG (5415, CST). Protein G/A-Sepharose^{TM} 4B (82085), Lipofectamine^{TM} 2000 (11668019) and Trizol (15596026) were purchased from ThermoFischer Scientific. Mag-Beads GST Fusion Protein Purification (C650031) and Mag-Beads His-Tag Protein Purification (C650033) were purchased from Sangon Biotech (Shanghai, China). Immobilon-P^{SQ} Transfer Membrane (ISEQ00010) and Immobilon Crescendo Western HRP substrate (WBLUR0500) were purchased from Millipore. MG-132 (S2619), Mebendazole (S4610) and PEG300 (S6704) were purchased from Selleck. Cycloheximide (2112) was purchased from CST. DMSO (D8148), Triton™ X-100 (2315025) and MTT (475989) were purchased from Sigma-Aldrich. The Dual-lumi^{TM} Luciferase Reporter Gene Assay Kit (RG088S) was purchased from Beyotime (Nanjing, China). The

SureScript™ First-Strand cDNA Synthesis Kit (QP056) and BlazeTaq™ SYBR® Green qPCR Mix 2.0 (QP031) were purchased from GeneCopoeia (Shanghai, China). The VectaFluor™ Horse Anti-Rabbit IgG, DyLight® 594 Antibody Kit (DI-1794), VectaFluor™ Horse Anti-Mouse IgG, DyLight® 488 Antibody Kit (DI-2488) and VECTASTAIN® ABC-HRP Kit (PK-4001) were purchased from Vector Laboratories. Crystal violet (C8470) was purchased from Solarbio (China). Annexin V- APC /7-AAD apoptosis kit (559763) was purchased from BD Biosciences.

Plasmids

The lentiviral vector pLKO.1 expressing USP5 shRNA or scramble non-target shRNA were constructed by Genechem (Shanghai, China), and verified by DNA sequencing. The target sequences of shRNA against CDS and 3'UTR of USP5 mRNA were 5'-GACCACACGATTTGCCTCATT-3', and 5'-CCTGTCTGTAAGGAGACTTTG-3', respectively. The plasmid expressing full length, deletion mutant or C335A mutant USP5 with Flag tag was kindly provided by Prof. Chengjiang Gao (Shandong University, China) [3]. The pcDNA3.1 expressing full length and deletion mutant EphA2 with Myc tag, and plasmid expressing HA-Ub, HA-UbK48 or HA-UbK63 have been described previously by us [4]. The USP5 promoter reporter plasmid GV238-USP5(-2000 to -30bp)-Luc, and its control plasmid GV238-Luc were constructed by Genechem (Shanghai, China) and verified by DNA sequencing. The vector pET-28α expressing human USP5 with His tag and the vector pGEX-4T-1 expressing EphA2 (662-1011aa) with GST-tag were constructed by Zoonbio Biotechnology (Nanjing, China) and verified by DNA sequencing.

Animal experiments

Female nude mice (BALB/c) that were four-week old were obtained from the Experimental Animal Center of Central South University and maintained in

pathogen-free conditions. For testing the effects of USP5/EphA2 axis on NPC cell radiosensitivity, 5×10^6 5-8F NPC cells with stable USP5 knockdown, 5-8F NPC cells with stable USP5 knockdown and EphA2 overexpression and scramble non-target shRNA control (shNC) cells in 100 μL serum-free medium were injected subcutaneously into nude mice respectively. Seven days after the inoculation (approximately 50 mm^3 of tumors), a 6Gy dose of ionizing radiation was delivered to the tumor (once daily for continuous 2 days), and the rest of the mouse was shielded with 10-mm lead blocks. Ionizing radiation was delivered using an experimental x-ray 225 irradiator (PXI, USA). Control mice received sham radiation.

For testing the radiosensitization effect of MBZ on NPC cells, 5×10^6 5-8F NPC with stable USP5 or EphA2 overexpression (OE) and their vector control cells in 100 μL serum-free medium were injected subcutaneously into nude mice respectively. Seven days after the inoculation, tumor-bearing mice received MBZ (5 mg/kg once daily for continuous 7 days) and/or a 6Gy dose of ionizing radiation (once daily for 2 continuous days). MBZ was prepared fresh daily by first dissolving it in DMSO and then suspending it in a 1% Cremophor/water mixture before administration. Control mice were treated with the equivalent DMSO/1% Cremophor/water solution. The specific schematic view of the treatment plans was described in the Figure 6D.

Tumor sizes were measured using an electronic caliper daily, and tumor volume was calculated using the formula (length$\times$width^2/2). The mice were euthanised at 12 days after initial treatment, their tumor were harvested and weighted using double-blinded evaluation. tumor tissues were fixed with 4% paraformaldehyde and embedded in paraffin for immunohistochemical staining.

GST pull-down assay

The GST pull-down assay was performed to detect USP5 directly interacting

with EphA2 as described previously by us [4]. Briefly, pET-28α expressing human USP5 with a His tag and pGEX-4T-1 expressing EphA2 with a GST tag were transfected into *E. coli* (TSC-E01, Tsingke biotechnology), respectively. GST-EphA2 fusion protein was purified using the Mag-Beads GST Fusion Protein Purification (C650031, Sangon Biotech) and the histidine (His)-USP5 fusion protein was purified using the Mag-Beads His-Tag Protein Purification (C650033, Sangon Biotech) according to the manufacturer's instructions. 20 μg GST or GST-EphA2 fusion protein was immobilized in 100 μL glutathione agarose and equilibrated at 4 °C for 4 hours. 20 μg His-USP5 fusion protein was added to 20 μg GST-EphA2 or GST immobilized on glutathione agarose and incubated in GST pull-down buffer at 4 ℃ overnight. After washing with PBS 5 times, the bound proteins were dissolved in 2×SDS loading buffer (P0015B, Beyotime), separated by SDS-PAGE, and subjected to immunoblotting with antibodies against GST (1:1000 dilution) or His (1:1000 dilution).

Western blot

Western blot was performed to detect the expression of proteins in the indicated NPC cells as described previously by us [5, 6]. Briefly, proteins were extracted from cells using RIPA lysis buffer. An equal amount of protein in each sample was subjected to SDS-PAGE separation, followed by blotting onto a PVDF membrane. After blocking in 5% defatted milk powder diluted in TBST, blots were incubated with primary antibodies described as the following: USP5 (1:100 dilution), EphA2 (1:100 dilution), GAPDH (1:2000 dilution), Ubiquitin (1:1000 dilution), HA-tag (1:2000 dilution), Flag-tag (1:1000 dilution), or Myc-tag (1:1000 dilution) overnight at 4 ℃, followed by incubation with HRP anti-rabbit IgG (1:2000 dilution) or HRP anti-mouse IgG (1:2000 dilution) for 2 hours at room temperature. The signal was

visualized with an enhanced chemiluminescence detection reagent (Roche).

Quantitative real-time (qRT)-PCR

QRT-PCR was performed to detect the expression of USP5 and EphA2 in NPC cells with stable knockdown of USP5, NPC cells treated with MBZ and their respective control cells. Briefly, total RNA was extracted from the indicated cells using Trizol reagent. 2μg of total RNA was reverse-transcribed into cDNA using SureScript^{TM} First-Strand cDNA Synthesis Kit (QP056, GeneCopoeia) according to the manufacturer's instruction. The reverse-transcribed products were amplified by real-time PCR using BlazeTaq^{TM} SYBR$^{®}$ Green qPCR Mix 2.0 (QP031, GeneCopoeia) according to the manufacturer's instruction. The expression of USP5 and EphA2 was quantitated using the 2^{-DDCt} method against GAPDH for normalization. The primers used are presented in Table S3. QRT-PCR was performed on the ABI Gene Amp PCR System 9700 (ABI).

Dual luciferase reporter assay

A dual luciferase reporter assay was performed to detect USP5 promoter activity as described previously [5]. Briefly, 1×10^5 NPC cells were plated into 6-well plates for 12 hours, and were transiently cotransfected with 1 μg of the GV238-USP5 promoter (-2000 to + 30bp)-Luc plasmid and 0.1 μg of the pRL-TK plasmid using Lipofectamine 2000. Cotransfection of GV238-Luc without USP5 promoter and the pRL-TK plasmid into cells served as a control. 12 hours after transfection, cells were treated with indicated concentrations of MBZ for 36 hours, and then cells were harvested. Both firefly luciferase and renilla luciferase activities were measured with Dual-lumi^{TM} Luciferase Reporter Gene Assay Kit (RG088S, Beyotime) according to the manufacturer’s instruction, and USP5 reporter activity was estimated using a GloMax$^{®}$ 96 Microplate luminometer (Promega).

Immunofluorescent staining

Immunofluorescent staining was performed to detect the subcellular location of USP5 and EphA2 and γH2AX expression as described previously by us [4]. Briefly, cells were plated into chamber slides (Millipore), fixed in 4% paraformaldehyde, permeabilized in 0.2% Triton X-100, and incubated with mouse anti-EphA2 antibody (1:100 dilution), rabbit anti-USP5 antibody (1:1000 dilution; A4202, abclonal) or rabbit anti-γH2AX antibody (1:500 dilution) at 4 ℃ overnight, followed by incubation with DyLight® 488 anti-mouse IgG or DyLight® 594 anti-rabbit IgG for 1 hour at room temperature. Nuclei were counterstained with DAPI. Images were captured using an inverted confocal fluorescent microscope (LEICA TCS SP8), and γH2AX foci per cell were calculated.

Immunohistochemistry and staining evaluation

Immunohistochemistry and staining evaluation of USP5, EphA2, cleaved caspase 3, γH2AX and Ki67 were performed on the formalin-fixed and paraffin-embedded tissue sections as described previously by us [7]. Briefly, tissue sections were deparaffinized in xylene, rehydrated through graded alcohol, and treated with an antigen retrieval solution (10 mmol/L sodium citrate buffer; pH 6.0). The sections were immersed in 3% hydrogen peroxide for 10 minutes to block endogenous peroxidase activity. To block nonspecific binding of antibody, the sections were preincubated with 10% nonimmune goat serum (Vector Laboratories) at room temperature for 15 minutes. Subsequently, the sections were incubated with USP5 antibody (1:50 dilution) or EphA2 antibody (1:50 dilution), cleaved caspase 3 antibody (1:400 dilution) or γH2AX antibody (1:500 dilution) or Ki67 antibody (1:100 dilution) overnight at 4 °C, and then incubated with biotinylated secondary antibody (1:400 dilution, Vector Laboratories) followed by avidin–biotin peroxidase

complex (Vector Laboratories) at room temperature for 30 minutes, and stained with DAB (3,3-diaminobenzidine; SK-4100, Vector Laboratories). Finally, tissue sections were counterstained with hematoxylin. In negative controls, primary antibodies were replaced with a normal mouse (1:100 dilution; 3900, CST) or rabbit IgG (1:100 dilution; 5415, CST).

Immunohistochemical staining was assessed and scored by two independent pathologists who were blinded to the clinic pathological data. Discrepancies were resolved by consensus. Positive reactions were defined as those showing brown signals in the cytoplasm and/or cytomembrane. Staining intensity was categorized: absent staining as 0, weak as 1, moderate as 2, and strong as 3. The percentage of stained cells (examined in at least 500 cells) was categorized as unstaining = 0, <30% stained cells =1, 30-60% = 2, and >60% = 3. Staining score (ranging from 0 to 6) for each tissue was calculated by adding the area and the intensity scores. A combined staining score of ≤3 was considered as low expression and >3 was considered as high expression.

Clonogenic survival assay

A clonogenic survival assay was performed to detect *in vitro* cell sensitivity to ionizing radiation as previously described by us [1, 2]. Briefly, cells were seeded into 6-well plates at a density of 2000 cells per well and cultured for 12 hours, and then were treated with indicated concentrations of mebendazole (MBZ) for 48 hours and/or indicated doses of ionizing radiation. 12 days after initial treatment surviving colonies were stained with 0.5% crystal violet and counted under a microscope. The surviving fraction (SF) of the irradiated cells was calculated using single-hit multi-target model equation: $SF = 1- (1- e- D/D0)^n$, in which D is radiation dose, D0 is mean lethal dose, and n is extrapolation number. Sensitive enhancement ratio (SER) was used to

evaluate the radiosensitizing effect of MBZ, which is defined as D0 value of control group divided by D0 value of MBZ treatment group.

Flow cytometry analysis of cell apoptosis

Cell apoptosis was detected using Annexin V-APC/7-AAD apoptosis kit as previously described by us [5]. Briefly, cells were treated with indicated concentration of MBZ and/or 4Gy ionizing radiation. 48 hours after treatment, cells were harvested and washed 1 time with ice-cold PBS. The cells were resuspended in annexin-binding buffer at 1×10^6 cells/mL. Then 5 μL of Annexin V-APC and 10 μl of 7-AAD were added per 100 μL of cell suspension. Cells were incubated at room temperature in dark for 10 minutes, and 400 μL of annexin-binding buffer was added. The cells were kept on ice until ready for analysis by a Dxp Athena flow cytometry (NL3000, Cytek).

MTT assay

MTT assay was performed to determine the IC50 (half maximal inhibitory concentration) of mebendazole (MBZ) on NPC cell proliferation. Briefly, 1×10^4 NPC cells were seeded in 96-well plates and cultured for 12 hours, and then 0.25, 1.25, 2.5, 5, 7.5, 10, 15, 20 μM (final concentration) of MBZ were added to cells. 24 hours after MBZ treatment, 20 μL of MTT solution (5mg/mL) was added into each well, followed by 4 hours of incubation. The medium was removed, and 150 μL of DMSO (sigma-Aldrich) was added to each well for 10 minutes of incubation, and the absorbance of each well was read by a spectrometer (Epoch, Biotek) at 490 nm. Three independent experiments were performed in triplicate.

Evaluation of USP5 and EphA2 as a biomarker for predicting NPC response to radiotherapy

The USP5 and EphA2 proteins were individually and, as a panel, assessed for their ability to discriminate between radiosensitive and radioresistant NPC patients by

evaluating their receiver operating characteristic (ROC) curve based on the immunohistochemistry scores described by us [1]. Briefly, we built a logistic regression model and conducted ROC curve analyses to evaluate overall predictive power of individual and the combined two proteins. The optimal cut point was determined for each protein by identifying the value that yielded the maximum corresponding sensitivity and specificity. ROC curves were then plotted based on the set of optimal sensitivity and specificity values. The area under the curve and other attributes were computed through the numerical integration of the ROC curves. Sensitivity, specificity, positive predictive value, and negative predictive value of the two proteins were calculated individually and as a panel. A two-sided $P < 0.05$ was considered significant.

References

1. Feng XP, Yi H, Li MY, Li XH, Yi B, Zhang PF, et al. Identification of biomarkers for predicting nasopharyngeal carcinoma response to radiotherapy by proteomics. Cancer Res. 2010; 70:3450-62.

2. Qu JQ, Yi HM, Ye X, Zhu JF, Yi H, Li LN, et al. MiRNA-203 reduces nasopharyngeal carcinoma radioresistance by targeting IL8/AKT signaling. Mol Cancer Ther. 2015; 14:2653-64.

3. Cai B, Zhao J, Zhang Y, Liu Y, Ma C, Yi F, et al. USP5 attenuates NLRP3 inflammasome activation by promoting autophagic degradation of NLRP3. Autophagy. 2022; 18:990-1004.

4. Feng J, Lu SS, Xiao T, Huang W, Yi H, Zhu W, et al. ANXA1 Binds and Stabilizes EphA2 to Promote Nasopharyngeal Carcinoma Growth and Metastasis. Cancer Res. 2020; 80:4386-98.

5. Xiao D, Zeng T, Zhu W, Yu ZZ, Huang W, Yi H, et al. ANXA1 Promotes Tumor Immune Evasion by Binding PARP1 and Upregulating Stat3-Induced Expression of PD-L1 in Multiple Cancers. Cancer Immunol Res. 2023; 11:1367-83.

6. Xiang YP, Xiao T, Li QG, Lu SS, Zhu W, Liu YY, et al. Y772 phosphorylation of EphA2 is responsible for EphA2-dependent NPC nasopharyngeal carcinoma growth by Shp2/Erk-1/2 signaling pathway. Cell Death Dis. 2020; 11:709.

7. Yi HM, Yi H, Zhu JF, Xiao T, Lu SS, Guan YJ, et al. A five-variable signaturepredicts radioresistance and prognosis in nasopharyngeal carcinoma patientsreceiving radical radiotherapy. Tumour Biol. 2016; 37:2941-49.

Table S1. The clinicopathological characteristics of 119 nasopharyngeal carcinoma patients

Parameters	Number of patients	Percent
**Age(y)**		
≤45	61	51.26 %
＞45	58	48.74 %
**Gender**		
Male	85	71.43 %
Female	34	28.57 %
**Smoking status**		
Current/former	57	47.90 %
Never	62	52.10 %
**Clinical TNM stage**		
I-II	43	36.13 %
III-IVa	76	63.87 %
**Primary tumor (T) stage**		
T1-2	55	46.22 %
T3-4	64	53.78 %
**Lymph node (N) metastasis**		
N0	27	22.69 %
N1-3	92	77.31 %
**Radiotherapeutic response**		
Radiosensitive	89	74.79 %
Radioresistant	30	25.21 %

Table S2. Correlations between the two protein expression and clinicopathological characteristics in NPC (n=119)

Variables	N	USP5			EphA2		
		Low	High	*p*	Low	High	*p*
**Age (y)**				0.270			0.098
<45	61	31	30		33	28	
≥45	58	23	35		22	36	
**Gender**				0.547			0.223
Male	85	37	48		36	49	
Female	34	17	17		19	15	
**Primary tumor(T) stage**				0.068			0.101
T1-2	55	30	25		30	25	
T3-4	64	24	40		25	39	
**Lymph node(N) metastasis**				0.048			0.008
N0	27	17	10		19	8	
N1-3	92	37	55		36	56	
**Clinical TNM stage**				0.002			＜0.001
I-II	43	28	15		30	13	
III-IVa	76	26	50		25	51	
**Radiation response**				0.039			0.031
Radiosensitive	89	45	44		46	44	
Radioresistant	30	9	21		9	21	
**Median DFS (months)**				＜0.001			＜0.001
		35.41± 9.72	23.26± 9.98		34.67± 10.44	23.71± 10.15	
**Median OS (months)**				＜0.001			＜0.001
		48.58± 11.60	34.17±1 0.95		47.88± 12.72	34.53± 11.20	

Chi-square test, $p < 0.05$ was considered statistically significant.

Table S3. The primers used for the amplification of the genes by qRT-PCR

No.	Gene name	GenBank Accession No.	Primer sequence
1	USP5	NM_001098536	F: 5’ -CGGATTTGACCTTAGCG-3’
			R: 5’ -CTGCCATCGAAGTAGCG-3’
2	EphA2	NM_004431	F: 5’ -TGGCTCACACACCCGTATG-3’
			R: 5’ -GTCGCCAGACATCACGTTG-3’
3	GAPDH	NM_002046	F: 5’ TGACTTCAACAGCGACACCCA-3’
			R: 5’ -CACCCTGTTGCTGTAGCCAA-3’

Table S4. Receiver operating characteristics from immunohistochemistry scores of the two proteins individually and as a panel

Proteins	Sensitivity	Specificity	PPV	NPV	AUC
USP5	0.69	0.73	0.72	0.75	0.70
EphA2	0.66	0.76	0.70	0.78	0.72
Risk Model	0.86	0.78	0.81	0.83	0.85

PPV: Positive predict value; NPV: Negative predict value; AUC: area under the curve.

Table S5. Univariate and cox multivariate analyses of prognostic factors for overall and disease-free survival (N =119)

Variables	Disease free survival (DFS)				Overall survival (OS)			
	Univariate analysis		Multivariate analysis		Univariate analysis		Multivariate analysis	
	P	**HR (95% CI)**	***P***	**HR (95% CI)**	***P***	**HR (95% CI)**	***P***	**HR (95% CI)**
**Age(y)**								
≤45 *vs.*＞45	0.465	0.824（0.490-1.386）	0.287	1.392（0.757-2.560）	0.708	0.905 （0.537-1.524）	0.083	1.751（0.929-3.300）
**Gender**								
Male *vs.* Female	0.435	1.280（0.689-2.380）	0.481	1.279（0.646-2.531）	0.359	1.336 （0.719-2.483）	0.320	1.425（0.709-2.868）
**Smoking status**								
Current/former *vs.* Never	0.072	1.621（0.957-2.745）	0.618	0.862（0.482-1.542）	0.124	1.512 （0.893-2.559）	0.281	0.723（0.401-1.303）
**Clinical TNM stage**								
Stage I-II *vs.* Stage III-IV	0.042	2.109（1.643-2.911）	0.010	3.541（1.355-9.253）	0.032	1.977 （1.566-2.684）	0.041	2.632（1.042-6.649）
**Primary tumor (T) stage**								
T1-2 *vs.* T3-4	0.218	1.387（0.824-2.332）	0.456	1.372（0.597-3.154）	0.187	1.228 （0.729-2.066）	0.592	1.256（0.546-2.885）
**Lymph node (N) metastasis**								
N0 *vs.* N1-3	0.337	0.715（0.361-1.417）	0.686	0.861（0.417-1.779）	0.200	0.640 （0.323-1.267）	0.397	0.723（0.342-1.531）
**USP5 level**								
Low *vs.* High	＜0.001	0.418（0.076-0.288）	0.001	0.124（0.035-0.436）	＜0.001	0.141 （0.072-0.276）	0.002	0.141（0.041-0.489）
**EphA2 level**								
Low *vs.* High	＜0.001	0.158（0.081-0.307）	0.014	0.404（0.131-0.628）	＜0.001	0.146 （0.075-0.286）	0.021	0.187（0.125-0.320）
**USP5/EphA2 level**								
High and High vs. High or High	0.025	2.437 （1.671-3.074）	0.033	1.682（1.595-2.306）	0.037	1.676 （1.316-2.811）	0.028	1.938（1.661-3.320）

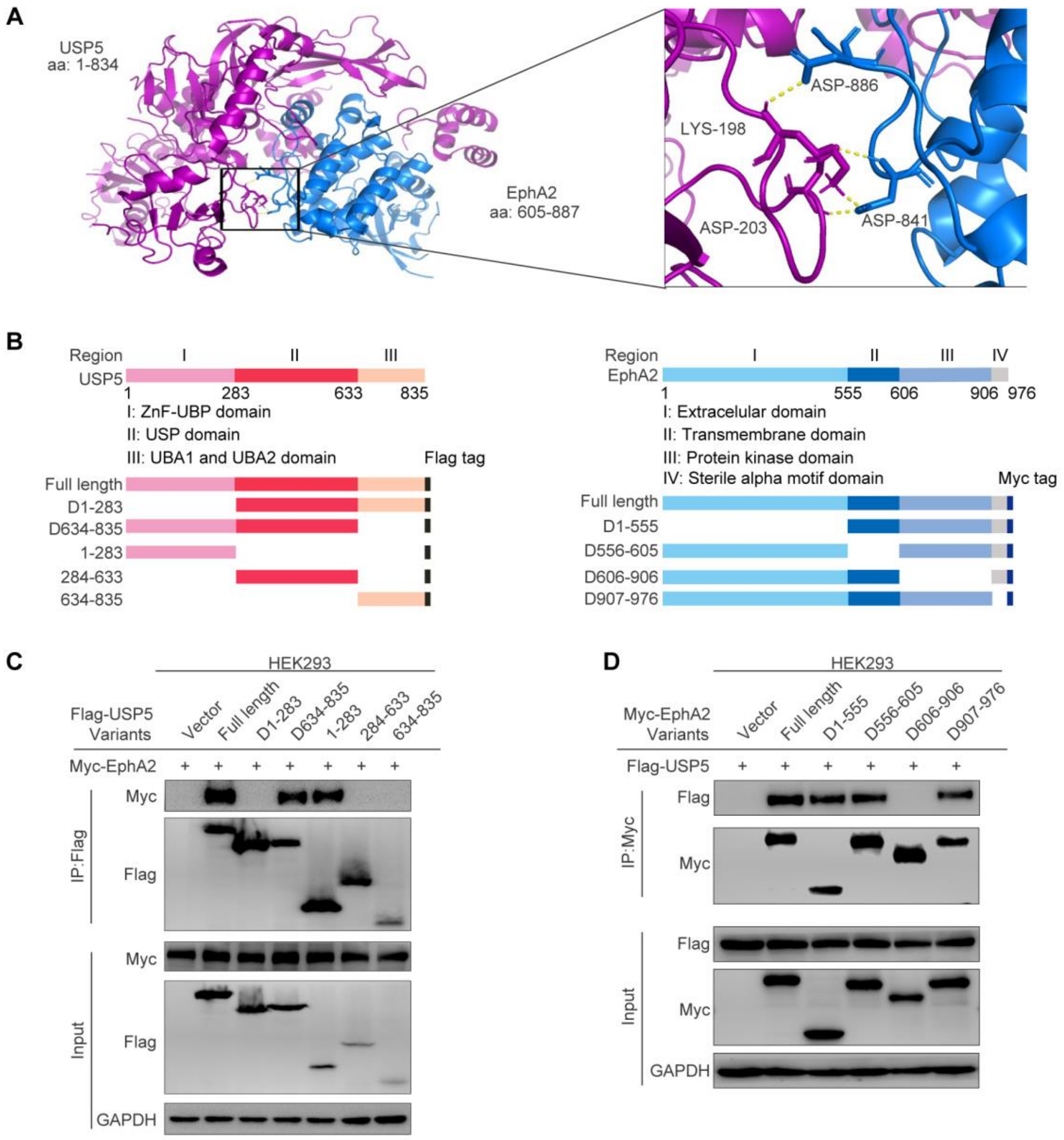

Figure S1. Mapping of the binding region of USP5 and EphA2. (**A**) Docking model for USP5 and EphA2 complex. The ZnF-UBP domain (LYS-198 and ASP-203) of USP5 (purple, Protein Data Bank code: 3IHP) binds to the tyrosine kinase domain (ASP-886 and ASP-841) of EphA2 (blue, Protein Data Bank code: 1MQB). (**B**) Diagrammatic representation of USP5, EphA2 and their deleted forms. The main regions of both proteins are indicated. Numbers indicate amino acid position within the sequence. D, deletion. (**C**) Co-IP showing the region of USP5 bound to EphA2. Total cell proteins from HEK293 cells transfected with the indicated constructs were subjected to immunoprecipitation with anti-Flag (USP5) antibody followed by

immunoblotting with antibodies against Myc (EphA2) or Flag (USP5). (**D**) Co-IP showing the region of EphA2 bound to USP5. Total cell proteins from HEK293 cells transfected with the indicated constructs were subjected to immunoprecipitation (IP) with anti-Myc (EphA2) antibody followed by immunoblotting with antibodies against Flag (USP5) or Myc (EphA2). IP, Immunoprecipitation; IB, Immunoblotting.

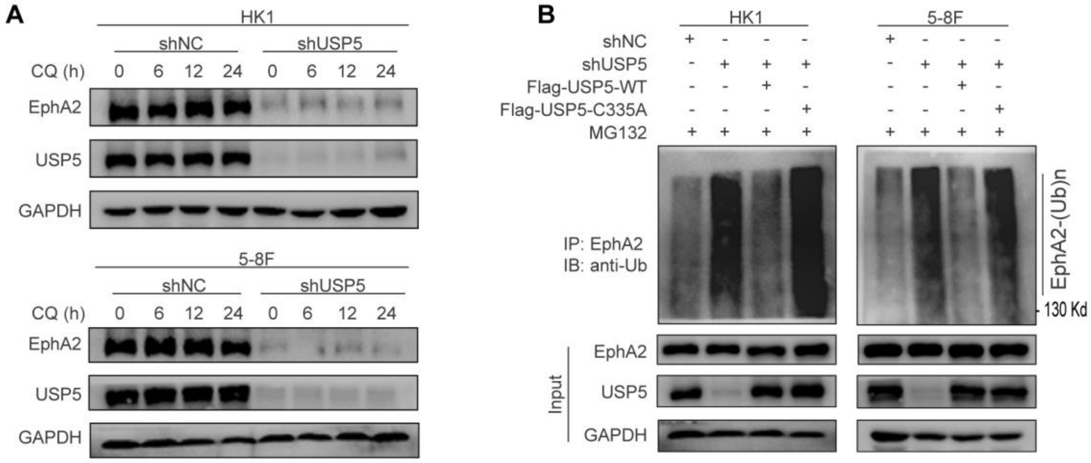

Figure S2. USP5 increases EphA2 protein stability by ubiquitin proteasome pathway in NPC cells. (**A**) The effect of chloroquine treatment on USP5 expression in the nasopharyngeal carcinoma (NPC) cells. Western blot showing that lysosome inhibitor chloroquine (CQ) could not reverse EphA2 protein levels in the USP5 knockdown HK1 and 5-8F NPC cells. Cells were treated with 25 μM CQ for indicated times, followed by immunoblotting with anti-EphA2 antibody. (**B**) The effect of catalytically inactive mutant USP5 (USP5-C335A) on EphA2 ubiquitination levels in NPC cells. USP5 knockdown HK1 and 5-8F NPC cells were transfected with wild-type EphA2 (USP5-WT) or USP5-C335A expression plasmid for 48 hours and treated with 10 μM MG132 for another 12 hours, and subjected to immunoprecipitation analysis with anti-EphA2 antibody followed by immunoblotting with anti-polyubiquitin antibody. shUSP5, knockdown of USP5 by shRNA; shNC, scramble shRNA negative control; IP, immunoprecipitation; IB, immunoblotting.

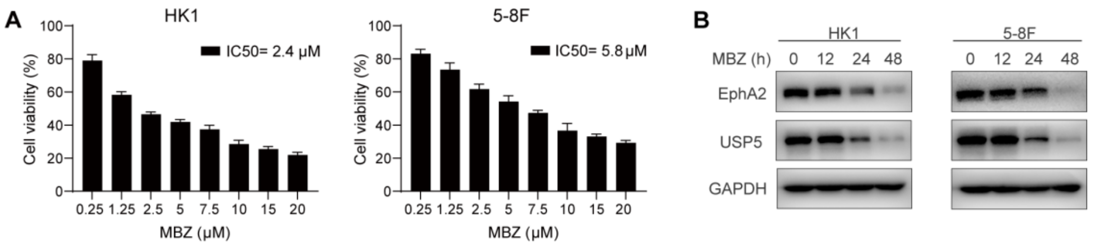

Figure S3. (**A**) The IC50 of Mebendazole (MBZ) on HK1 and 5-8F NPC cell proliferation. 1×10^4 NPC cells were seeded in 96-well plates and cultured for 12 hours, and then 0.25, 1.25, 2.5, 5, 7.5, 10, 15, 20 μM (final concentration) of MBZ were added to cells. 24 hours after MBZ treatment, MTT assay was performed to determine the IC50 of MBZ on NPC cell proliferation. (**B**) Western blot showing the expression levels of USP5 and EphA2 in the HK1 and 5-8F NPC cells treated with IC50 concentration of MBZ for indicated times.

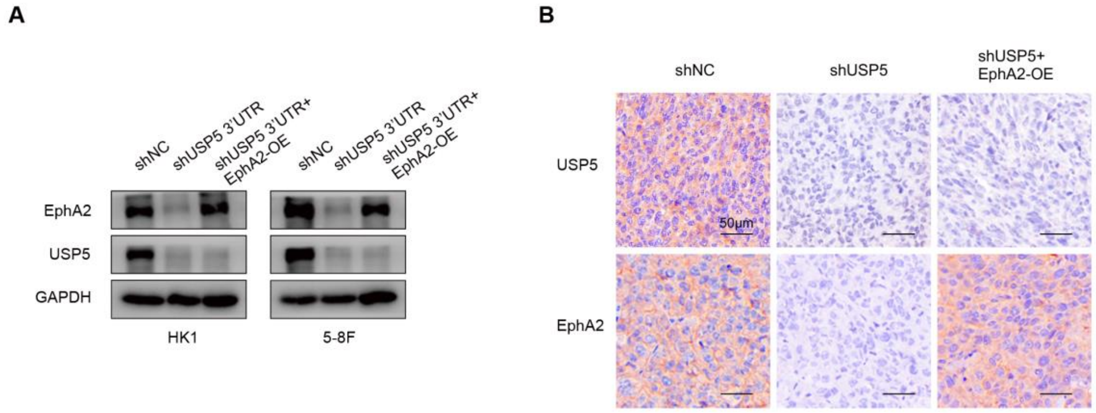

Figure S4. The expression level of USP5 and EphA2 in the established NPC cell lines and their xenografted tumors. (**A**) Western blot showing the expression levels of USP5 and EphA2 in the HK1 and 5-8F cell lines with USP5 knockdown or with USP5 knockdown and EphA2 overexpression, and their respective shNC control cells. (**B**) Representative immunohistochemistry (IHC) images of USP5 and EphA2 expression in the subcutaneous xenografted tumors of 5-8F NPC cells with USP5 knockdown, 5-8F NPC cells with USP5 knockdown and EphA2 overexpression, and

shNC control 5-8F cells. shUSP5, knockdown of USP5 by shRNA; EphA2-OE, EphA2 overexpression.

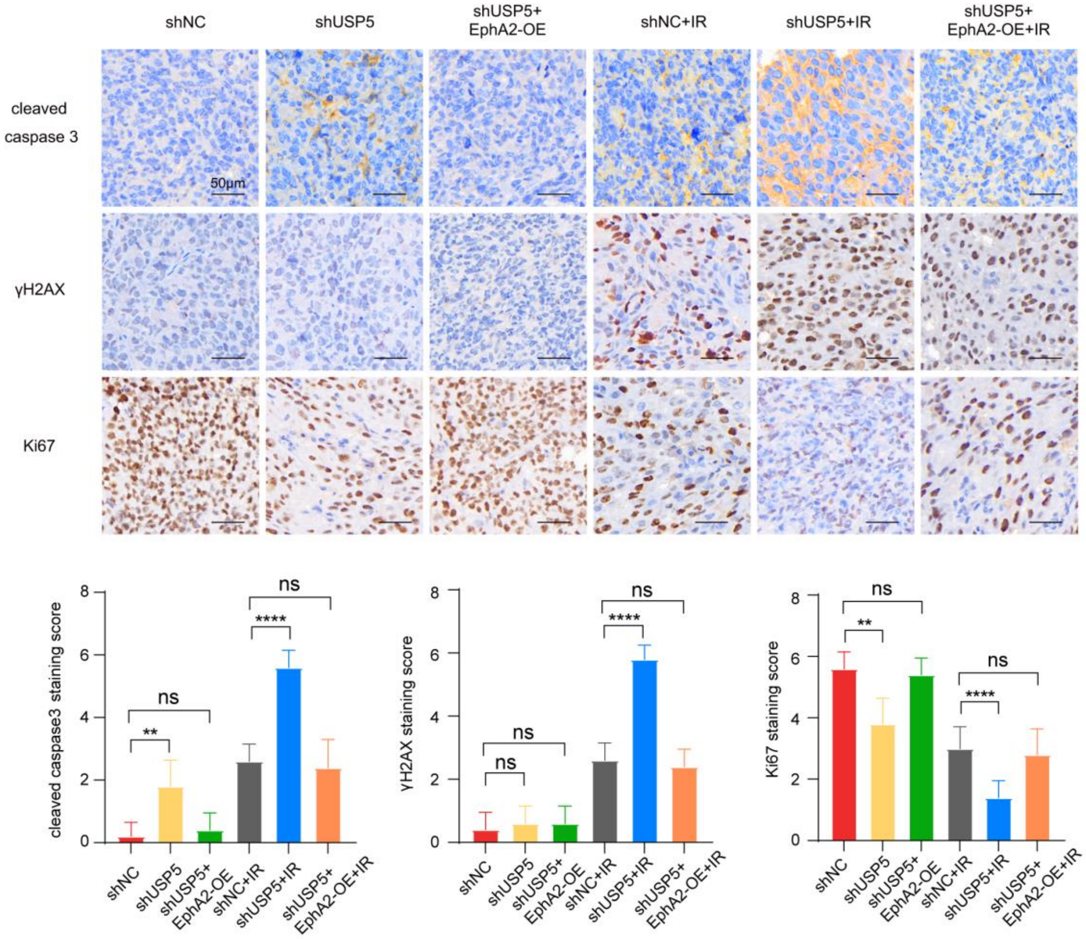

Figure S5. The effect of ionizing radiation on expressions of cleaved caspase 3, γH2AX, and Ki67 in the xenografted tumors of 5-8F cells with USP5 knockdown, 5-8F cells with USP5 knockdown and EphA2 overexpression, and shNC control 5-8F cells. Representative IHC images of cleaved caspase 3, γH2AX, and Ki67 expression in both ionizing radiation and no irradiation tumors are presented on the top, and quantitative data are presented on the bottom. Scale bars = 50 μm. The data represent the mean ± SD. **, $P < 0.001$; ****, $P < 0.0001$; ns, no significance. IR, ionizing radiation; shUSP5, knockdown of USP5 by shRNA; EphA2-OE, EphA2 overexpression.

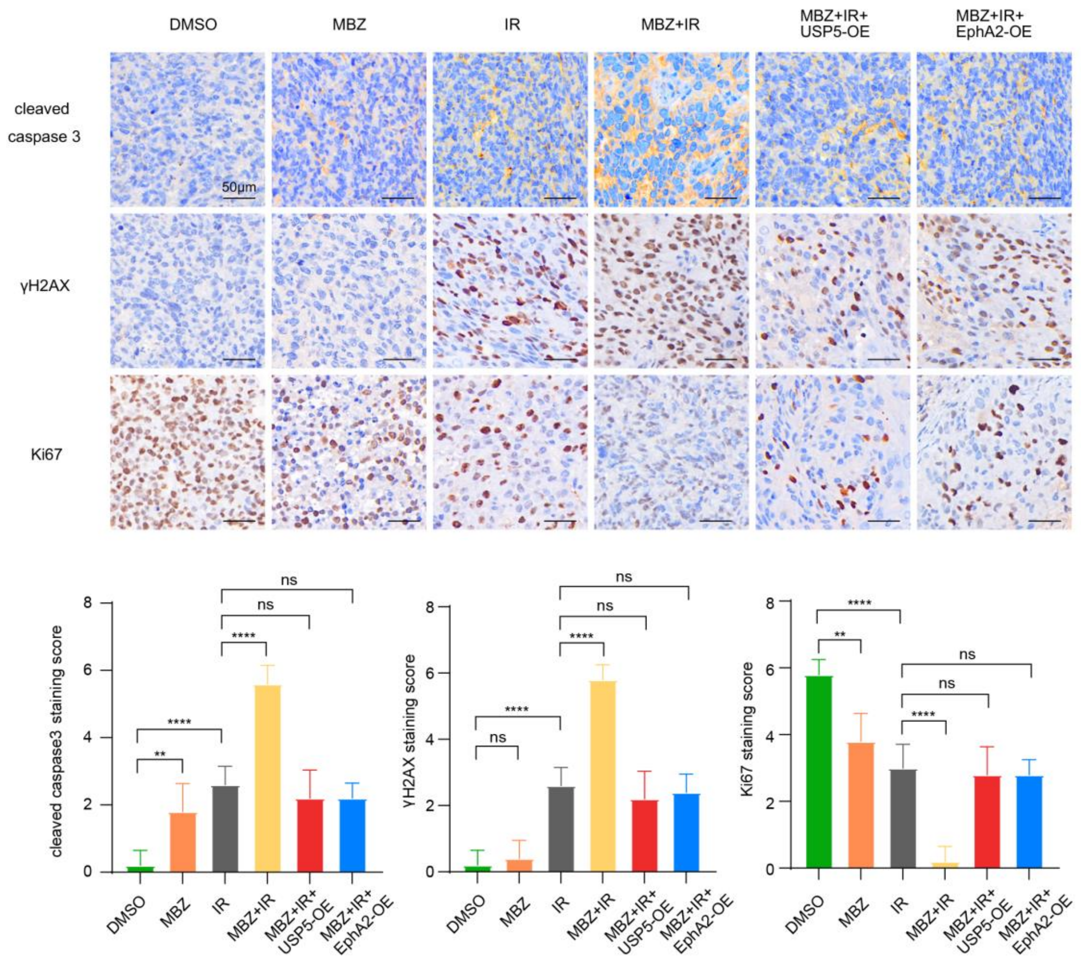

Figure S6. The Effect of MBZ or/and IR on expressions of cleaved caspase 3, γH2AX, and Ki67 in the xenografted tumors generated from 5-8F NPC cells. Representative IHC images of cleaved caspase 3, γH2AX, and Ki67 expression in the xenogrfted tumors treated with MBZ or/and IR are presented on the top, and quantitative data are presented on the bottom. Scale bars = 50 μm. The data represent the mean ± SD. **, *P* < 0.01; ****, *P* < 0.0001; ns, no significance. MBZ, mebendazole; IR, ionizing radiation; USP5-OE, USP5 overexpression; EphA2-OE, EphA2 overexpression.

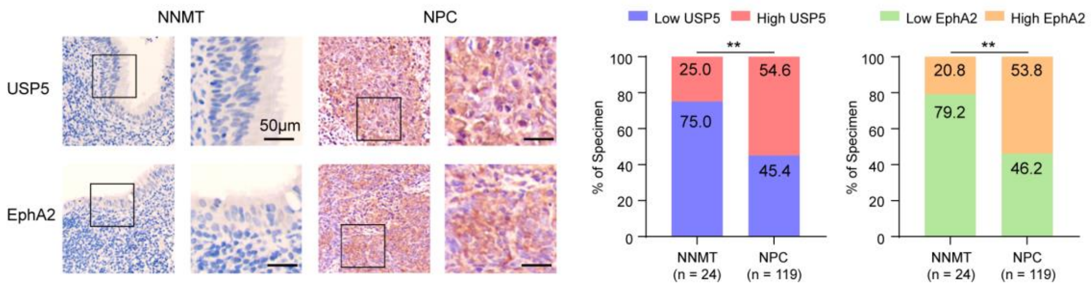

Figure S7. Expression level of USP5 and EphA2 in the NPC and normal nasopharyngeal mucosal tissues. Representative IHC images are shown on the left, and quantitative data are presented on the right. Scale bars = 50 μm. Statistical differences were determined by Chi-square (χ^2) test. **, *P*< 0.01. NNMT, normal nasopharyngeal mucosal tissue.

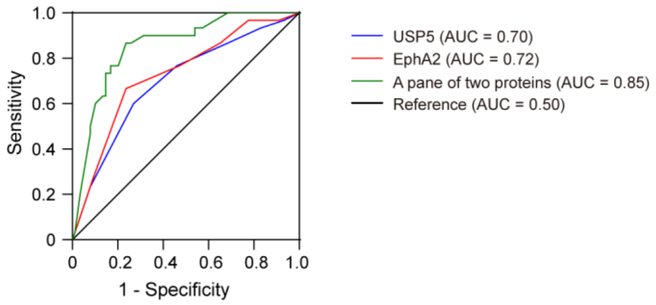

Figure S8. Receiver operating characteristic (ROC) curves of USP5 and EphA2 in discriminating radiosensitive and radioresistant NPC patients, individually and as a panel.
